# Preliminary Structure-Activity Relationship (SAR) of a Novel Series of Pyrazole SKF-96365 Analogues as Potential Store-Operated Calcium Entry (SOCE) Inhibitors

**DOI:** 10.3390/ijms19030856

**Published:** 2018-03-14

**Authors:** Camille D. Dago, Paul Le Maux, Thierry Roisnel, Christophe Brigaudeau, Yves-Alain Bekro, Olivier Mignen, Jean-Pierre Bazureau

**Affiliations:** 1 Institut des Sciences Chimiques de Rennes (ISCR), UMR CNRS 6226, Groupe CORINT, Université de Rennes 1 (UR1), Campus de Beaulieu, Bât. 10A, 263 Avesnue du Général Leclerc, CS 74205, 35042 Rennes CEDEX, France; deliko.dago@univ-rennes1.fr (C.D.D.); paul.lemaux@free.fr (P.L.M.); 2 Laboratoire de Chimie Bio Organique et de Substances Naturelles (LCBOSN), Université Nangui Abrogoua (UNA), Abidjan BP 802, Côte d’Ivoire; yvesalainb2014@gmail.com; 3 Institut des Sciences Chimiques de Rennes (ISCR), UMR CNRS 6226, Centre de Diffractométrie X (cdifx), Université de Rennes 1 (UR1), Campus de Beaulieu, Bât. 10B, 263 Avenue du Général Leclerc, CS 74205, 35042 Rennes CEDEX, France; thierry.roisnel@univ-rennes1.fr; 4 Laboratoire Canalopathies & Signalisation Calcique, Inserm U1227, Université de Bretagne Occidentale (UBO), 22 Avenue Camille Desmoulins, 29200 Brest CEDEX, France; christophe.brigaudeau@univ-brest.fr; 5 CalciScreen Platform, Université de Bretagne Occidentale (UBO), 22 Avenue Camille Desmoulins, 29200 Brest CEDEX, France; 6 S2Wave Platform, ScanMAT UMS 2001 CNRS, Université de Rennes 1 (UR1), Campus de Beaulieu, Bât. 10A, 263 Avenue du Général Leclerc, CS 74205, 35042 Rennes CEDEX, France

**Keywords:** pyrazole, SKF-96365, analogues, resolution, half-quantities, SOCE, B lymphocyte cell, SOCE inhibitor, Ca^2+^ signalling

## Abstract

From a series of (1*R*, 1*S*)-1[β-(phenylalkoxy)-(phenetyl)]-1*H*-pyrazolium hydrochloride as new analogues of SKF-96365, one has an interesting effect for endoplasmic reticulum (ER) Ca^2+^ release and store-operated Ca^2+^ entry (SOCE) (IC_50_ 25 μM) on the PLP-B lymphocyte cell line. A successful resolution of (±) 1-phenyl-2-(1*H*-pyrazol-1-yl)ethan-1-ol has been developed by using the method of “half-concentration” in the presence of (+)-(1*S*)- or (−)-(1*R*)-CSA.

## 1. Introduction

**Figure 1:**
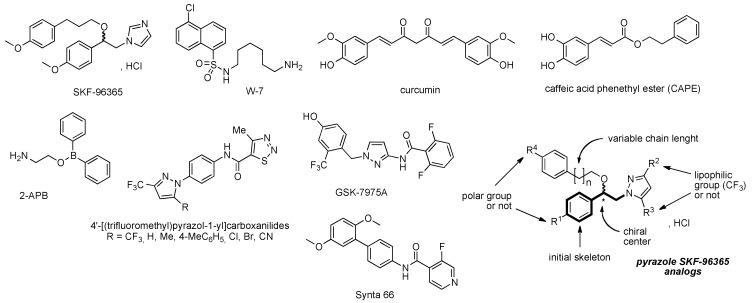
Structure of pharmacological inhibitors of calcium channels and structure–activity relationship (SAR) parameters of the pyrazole SKF-96365 analogues.

Cytoplasmic Ca^2+^ is very important for fundamental biological processes; this includes cell proliferation, apoptosis, migration, and gene expression [1]. The increase of cytoplasmic Ca^2+^ concentration following many plasma membrane receptors is connected to the release of stored Ca^2+^ within the endoplasmic reticulum (ER) or sarcoplasmic reticulum (SR) associated to the influx of extra-cellular Ca^2+^ across the plasma membrane. Store-operated Ca^2+^ entry (SOCE) is the typical mechanism to generate Ca^2+^ signals that combines the intracellular and extra-cellular processes and represents one of the most common and ubiquitous Ca^2+^ influx routes in non-excitable cells [2,3,4]. SOC entry is mediated by calcium selective channels such as the archetypal calcium-release-activated calcium (CRAC) channels in lymphocytes, which are typically supported by Orai1 proteins and regulated by STIM1 (Stromal Interacting Molecule). STIM1 is a trans-membrane protein mainly located in the ER membrane acting as a calcium-sensing protein, whereas Orai1 is located in the plasmalemma forming the calcium-selective pore of the CRAC channel [5]. STIM and Orai are expressed in all tissues and therefore are of first important in the numerous cellular functions. Abnormal SOC channels activities cause several human diseases, such as breast cancer [6,7,8], inflammatory bowel diseases [9], thrombosis [10], and severe combined immunodeficiency (SCID) disorders [11], which leads to an increasing interest in developing small molecule compounds to regulate aberrant SOC and especially CRAC channels function [12]. The therapeutic potential of inhibiting CRAC currents (named *I_CRAC_*) has been established by the clinical use of calcineurin inhibitors (cyclosporine A and tacrolinus) to prevent rejection of organ transplants. Examination of literature showed that the first identified inhibitor of the CRAC channel was SKF-96365 or 1-{β-[3-(4-methoxy-phenyl)propoxy]-4-methoxyphenethyl}-1*H*-imidazole hydrochloride [13,14] in 1990 (Figure 1) and is still used as a tool for the probing of receptor-mediated Ca^2+^ entry processes [15] in non-excitable cells [16], but it is interesting to note that the synthesis of SKF-96365 was not detailed in academic literature and also not patented. Recent studies demonstrated that it strongly inhibits voltage-gated sodium current (*I_Na_*) in rat ventricular myocytes using the whole-cell patch voltage-clamp technique [17]. SKF-96365 SOCE inhibitor exhibited potent anti-neoplastic activity by inducing cell-cycle arrest and apoptosis in colorectal cancer cells (HCT-116 and HT29 cells) [18]. Effect of SKF-96365 was also observed on hERG current in HEK 293 cells in a concentration-dependent manner. These blocking properties were similar to those observed previously for hERG channels by the calmodulin inhibitor W-7 [19]. All together, these results suggest a low specificity of this compound for CRAC channels.

A variety of new small molecules blocking the CRAC channels have been identified and developed, i.e., curcumin and caffeic acid phenethyl ester (CAPE) in ORAI1/STIM-co-expressing HEK 293 cells [20], 4′-[(trifluoromethyl)pyrazol-1-yl]carboxanilides exhibiting high selectivity for the CRAC channel over the voltage-operated Ca^2+^ (VOC) channels [21], 2-APB with a concentration-dependent effect [22], GSK-7975A by altering the Orai pore geometry [23], Synta 66 with good selectivity for CRAC channels, and no effect for Ca^2+^ pumps and K^+^ channels and no interference with STIM1 aggregation [24].

In this context [25], we decided to examine the synthesis of SKF-96365 analogues bearing substituted (or not) pyrazole platforms and the other parameters for this structure-activity relationship (SAR) study are respectively the length of the chain in position Cβ and the presence (or not) of a methoxy polar group in *para*-position on the phenylethyl skeleton and on the phenylalkyloxy side chain. The generic phenylethyl skeleton is maintained for this SAR study. Effects of these analogues are also examined for endoplasmic reticulum (ER) Ca^2+^ release and SOCE on a B lymphocyte cell line [26].

## 2. Results and Discussion

### 2.1. Chemistry

**Scheme 1:**
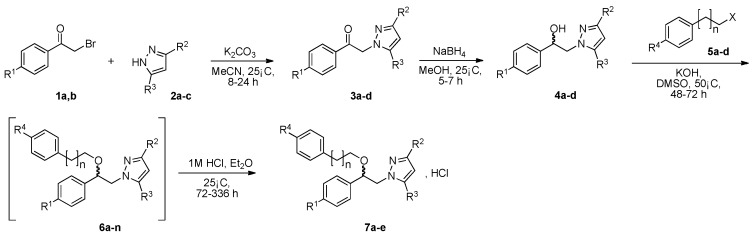
Synthesis of (1*R*, 1*S*) 1-[β-(phenylalkoxy)-phenethyl]-1*H*-pyrazolium hydrochloride **7a**–**e**.

These pyrazole analogues of SKF-96365 were prepared as shown in Scheme 1 using a modified method described in literature [27]. The first step involved reaction of 2-bromoacetophenone **1a** or 2-bromo-1-(4-methoxyphenyl)ethan-1-one **1b** with various pyrazoles **2** substituted by one or two trifluoromethyl groups (**2a**: pyrazole, **2b**: 3-trifluoromethylpyrazole, and **2c**: 3,5-*bis*-trifluoromethylpyrazole). The reaction was conducted with potassium carbonate in acetonitrile at room temperature. After work-up, the four desired *N*-substituted pyrazoles **3a**–**d** were obtained in yields ranging from 53% to 69% (Table 1). Reduction of the ketone function of **3a**–**d** was realized in methanol solution at 25 °C using sodium tetrahyborohydride during 5–7 h. The desired hydroxyl compounds **4a**–**b** were obtained after elimination of volatile compounds in vacuo, and simple treatment of the crude reaction mixture with deionized water afforded **4a**–**d** in good yields (82–98%) after crystallization.

In the next step, for the introduction of molecular diversity on Cβ hydroxyl group of **4** by *O*-alkylation, we used a series of alkyl halides **5** carrying various chains (*n* = 0 or 2) and methoxy group in *para*-position of the phenyl moiety (**5a**: 4-methoxybenzyl chloride; **5b**: 1-(2-bromoethyl)-4-methoxybenzene; **5c**: 1-(3-bromopropyl)-4-methoxybenzene; **5d**: 3-phenylpropyl bromide). For optimization of the reaction condition parameters, we developed a set of experiments which are the following: (i) the choice of the solvent (DMF, DMSO, MeCN, etc.); (ii) the choice of appropriate base (DIPEA, Et_3_N, KOH); (iii) the number of equivalents for the base; (iv) the reaction time; and (v) appropriate reaction temperature. We obtained good reproducibility (Table 2 for each product) when we applied a reaction time of 48 or 72 h (monitored by thin layer chromatography on silica with appropriate eluent for evaluation of consumption of the starting reagents), 2–5 equivalents of KOH, heating at 50 °C (during 48 or 72 h) after addition of alkyl halide **5** to produce the intermediate **6** which was not isolated (initial attempts to isolate it by flash chromatography on silica gel failed). After the addition of saturated brine to the crude reaction mixture (to solubilize the *N*-alkylated pyrazolium by-product), the intermediate **6** was extracted with dry diethyl ether and the collected extracts were directly treated with a commercial solution of 1 M HCl in ether. The desired salt **7** was collected by simple filtration after complete crystallization. It should be noted that this protocol yielded compounds **7a**–**e** in 10–33% (Table 1), on the contrary, attempts to prepare the lipophilic trifluoromethyl derivatives **7f**–**l** failed in spite of long crystallization times (168 and 336 h) at 4 °C and modification of the solvent for reaction (THF, dioxane).

**Figure 2:**
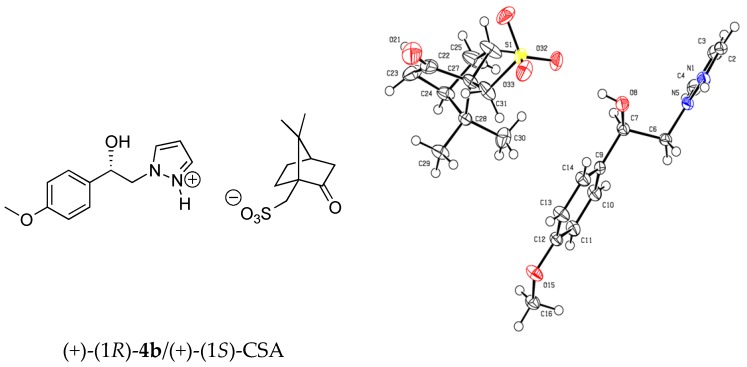
Ortep diagram of (+)-(1*R*) 1-(4-methoxyphenyl)-2-(1*H*-pyrazol-1-yl)ethan-1-ol **4b**/(+)-(1*S*)-10-camphorsulfonic acid obtained by X-ray diffraction.

**Scheme 2:**
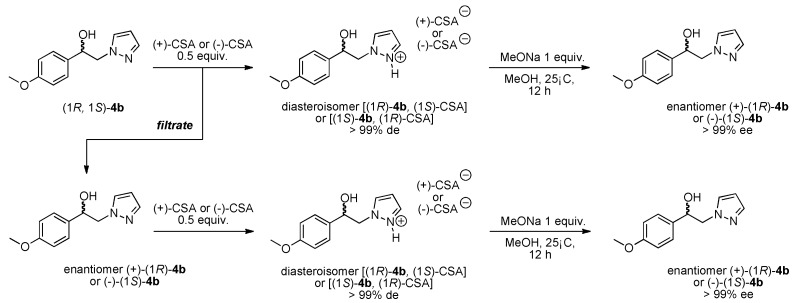
Resolution of (±)-(1*R*, 1*S*) 1-(4-methoxyphenyl)-2-(1*H*-pyrazol-1-yl)ethan-1-ol **4b** from (1*R*)-(−)-10-camphorsulfonic acid and (1*S*)-(+)-10-camphorsulfonic acid.

For this structure-activity relationship (SAR) study, we were also interested to evaluate the potential impact of chirality’s of the compounds **7** on SOC channels activities because our present protocol for preparation of these pyrazole SKF-96365 analogues produced a (±) racemic mixture. Based on this finding, our attention was attracted by the method of half-quantities [28] for resolution of our (±)-(1*R*, 1*S*)-1-(4-methoxyphenyl)-2-(1*H*-pyrazol-1-yl)ethan-1-ol **4b** mixture (Scheme 2). To find a simple and cost effective resolution procedure for (1*R*)-**4b** and (1*S*)-**4b**, we used commercial (+)-(1*S*)- and (−)-(1*R*)-10-camphorsulfonic acid (CSA). Treatment of (±)-(1*R*, 1*S*)-1-(4-methoxyphenyl)-2-(1*H*-pyrazol-1-yl)ethan-1-ol **4b** with 0.5 equivalent of (−)-(1*R*)-CSA in dry acetone caused the formation of a fine suspension for the first fraction of diastereomer (−)-(1*S*)-**4b**/(−)-(1*R*)-CSA (>85% de), which was recrystallized in dry acetone (52% isolated yield, >97% de). Then, a solution of this salt [(−)-(1*S*)-**4b**/(−)-(1*R*)-CSA] in dry acetone was submitted to diasteromeric enrichment by addition of 0.02 equivalent of (±) racemic **4b**. After work-up the pure (−)-(1*S*)-**4b**/(−)-(1*R*)-CSA was obtained in 48% yield (>99% de) as white powder with [α_D_] = −6.0 (*c* 1.0, MeOH) (Table 3). This protocol was also applied to (+)-(1*S*)-CSA for resolution of the (±) racemic **4b**, and after diastereomeric enrichment, the other diastereomer [(+)-(1*R*)-**4b**/(+)-(1*S*)-CSA, (>99% de)] was obtained in 51% yield with [α_D_] = +6.0 (*c* 1.0, MeOH). Crystal structures of the salt diastereomers were determined by X-ray crystal structure analysis, as example the diastereomer (+)-(1*R*)-**4b**/(+)-(1*S*)-CSA is shown in Figure 2.

Next, access to the free enantiomer (1*S*)-**4b** and (1*R*)-**4b** was operated by a simple neutralization of the respective diastereomers (−)-(1*S*)-**4b**/(−)-(1*R*)-CSA or (+)-(1*R*)-**4b**/(+)-(1*S*)-CSA using exactly 1 equivalent of MeONa in dry MeOH. After mixing during 12 h followed by elimination of volatile compounds in vacuo, the crude reaction mixture was treated with deionized water and the resulting insoluble enantiomer (−)-(1*S*)-**4b** or (+)-(1*R*)-**4b** precipitated and was collected by classical filtration. As can be seen in Table 3, pure enantiomers (−)-(1*S*)-**4b** and (+)-(1*R*)-**4b** were obtained respectively in isolated yields of 38 and 41% with [α_D_] = −10.0 (*c* 1.0, MeOH) for (−)-(1*S*)-**4b** (>99% ee) and [α_D_] = +10.0 (*c* 1.0, MeOH) for (+)-(1*R*)-**4b** (>99% ee).

During the resolution of the (±) racemic mixture **4b** by the “half-quantities” protocol with (−)-(1*R*)-CSA or (+)-(1*S*)-CSA and neutralization of the two diastereomers, all these compounds were submitted to chiral HPLC analysis with a Chiracel OJ-H column (250 × 4.6 i.d. mm) and a UV detector at 220 nm using hexane/*i*-PrOH as mobile phase with appropriate composition and flow rate.

For the (±) racemic mixture **4b**, we obtained only two peaks, the retention time of the first and second eluted enantiomers were measured respectively as 65.9 and 71.4 min (hexane/*i*-PrOH 96/4 *v*/*v*, flow rate = 0.6 mL/min). The quality of the results obtained by the “half-quantities” and neutralization methods were confirmed by the presence of only one peak for the two diastereomers (−)-(1*S*)-**4b**/(−)-(1*R*)-CSA, (+)-(1*R*)-**4b**/(+)-(1*S*)-CSA and the two enantiomers (−)-(1*S*)-**4b** and (+)-(1*R*)-**4b**. These data were summarized in Table 3 (the corresponding chromatograms were reported in Supplementary Materials).

**Scheme 3:**
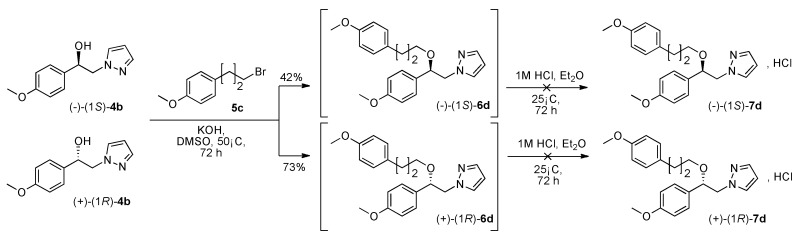
Synthesis of 1-[β-((4-methoxyphenyl)-(3-(4-methoxyphenyl)propoxyethyl)]-1*H*-pyrazole (−)-(1*S*)-**6d**, (+)-(1*R*)-**6d** and assays to their hydrochloride derivatives.

With the two pure enantiomers (−)-(1*S*)-**4b**, (+)-(1*R*)-**4b** on hand, we were stimulated to prepare potentially the compounds (−)-(1*S*)-**7d** and (+)-(1*R*)-**7d** with the alkyl halide 1-(3-bromopropyl)-4-methoxybenzene **5c** according to Scheme 2. We applied the protocol used initially for the preparation of racemic (1*R*, 1*S*)-**7d** using 5 equivalents of KOH in solution of DMSO at 50 °C during 72 h from (−)-(1*S*)-**4b** or (+)-(1*R*)-**4b** and 4-(3-bromopropyl)-4-methoxybenzene **5c** (Scheme 3). Progress of the transformation and consumption of the starting reagents were monitored by ^1^H NMR and also by thin layer chromatography on silica plates. After extraction with dry Et_2_O and treatment of the collected extracts with a solution of 1 M HCl, to our surprise, we did not observe precipitation of the desired (−)-(1*S*)-**7d** and (+)-(1*R*)-**7d**. We tried to change the solvent for extraction (dioxane as example) but this modification was not well suited for crystallization at room temperature even for an additional day at 4 °C.

### 2.2. Biology

**Figure 3:**
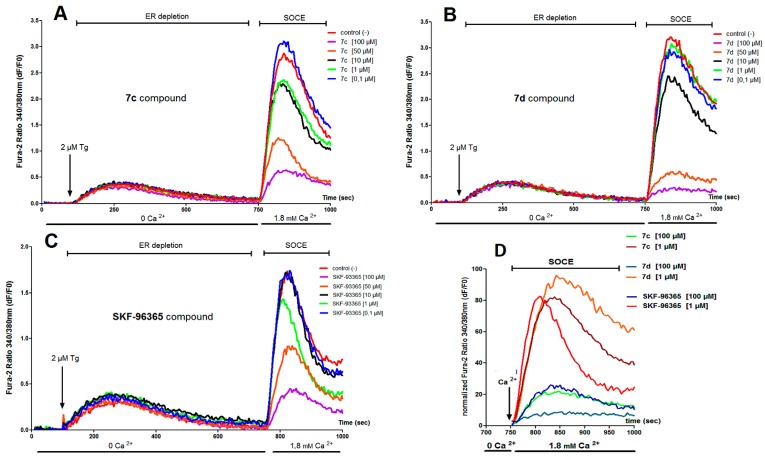

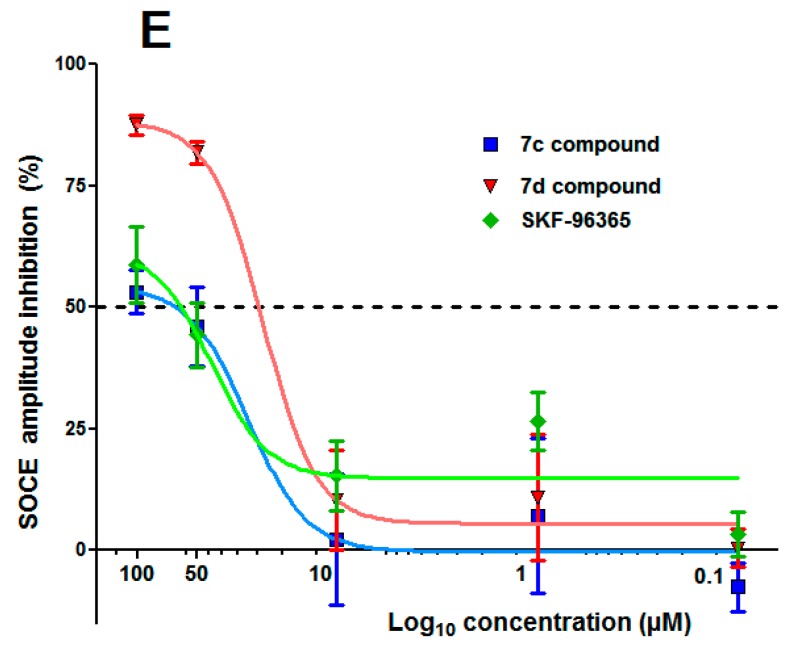
Effect of compounds **7c**, **7d**, and SKF-96365 compounds on ER Ca^2+^ release and store-operated Ca^2+^ entry (SOCE). (**A**–**D**) showed typical representative traces of intracellular Ca^2+^ concentration variations measured with Fura-2 dye in PLP-B lymphocyte cell line when SOCE activation protocol is implemented. Tested compounds **7c**, **7d**, and SKF-96365 were added 3 min before recording intracellular Ca^2+^ level variation with the FlexStation 3™ plate reader. Endoplasmic reticulum (ER) Ca^2+^ content is first totally depleted in Ca^2+^ free medium by injection of 2 μM Thapsigargin (Tg) and SOCE is next measured by addition of 1.8 mM Ca^2+^ in the extracellular medium; (**D**) Representative traces of the SOCE Ca^2+^ signals recorded after ER Ca^2+^ stores releases with Tg in cells treated with **7c**, **7d**, and SKF-96365 compounds (100 and 1 μM). SOCE amplitudes in the presence of the different drugs are normalized to vehicle control; (**E**) **7c**, **7d**, and SKF-936365 compounds inhibition dose-response curves: points represented the mean ±SEM of three different experiments and expressed as the percentage of SOCE Ca^2+^ entry amplitude inhibition on PLP-B Lymphocyte cell line normalized to vehicle control (100%).

The synthesized hydrochloride compounds **7a**–**c** as described above were evaluated for their ability on endoplasmic reticulum (ER) Ca^2+^ release and SOCE using the PLP-B lymphocyte cell line. For comparison, commercial SKF-96365 hydrochloride was used as reference. All compounds **7** and SKF-96365 were added 3 min before recording intracellular Ca^2+^ level variation in the presence of Fura-2 dye loaded on PLP-B lymphocyte cell. Fluorescence measurements were realized in Flex Station™ 3 microplate reader. Fura-2 loaded PLP-B lymphocyte cells were stimulated with 2 μM Thapsigargin (Tg) for 30 min and SOCE was measured after addition of 1.8 mM Ca^2+^ in the extracellular medium. Results are respectively summarized in Table 4 and also in Figure 3A–E. For this structure–activity relationship (SAR) study, we examined mainly the effect of MeO substituent on the *para*-phenyl group of the phenethyl-1*H*-pyrazolium skeleton and also on the phenylalkoxy chain of Cβ compared to the SKF-96365 reference.

**Figure 4:**
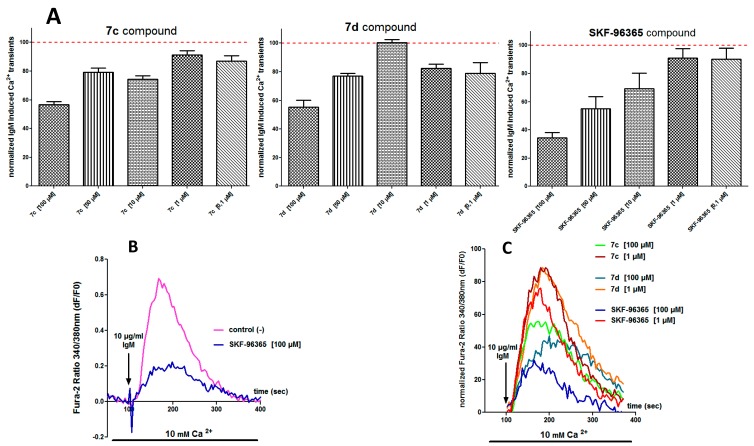
Effect of **7c**, **7d**, and SKF-96365 compounds on BCR (B Cell Receptor) induced Ca^2+^ responses in B cells stimulated with IgM in the presence of extracellular Ca^2+^. (**A**) histograms represent the average inhibition of IgM-induced Ca^2+^ transients in PLP cells treated with five different concentrations of **7c**, **7d** and SKF-96363 compounds (100, 50, 10, 1 and 0.1 µM). Data are normalized to vehicle control (100%) and represented as the mean ± SEM of 3 different experiments; (**B**,**C**) panels show typical representative traces of intracellular Ca^2+^ concentration variations measured with Fura-2 dye in PLP cells with BCR activation stimulation by IgM in the presence or absence of the different tested drugs. Tested compounds **7c**, **7d**, and SKF-96365 were added 3 min before recording intracellular Ca^2+^ level variation. BCR-induced Ca^2+^ responses are measured in 10 mM Ca^2+^ extracellular medium in cells stimulated with 10 µg/mL IgM; (**B**) Representative traces obtained in control (−) conditions or in the presence of SKF-696365 (100 µM); (**C**) Representative traces normalized to control conditions in cells treated with **7c**, **7d**, and SKF-96365 compounds (100 and 1 µM).

Examination of Table 1 and also dose response curves (Figure 3E) showed that compound **7d** (IC_50_ 25 μM) was a better potent SOCE inhibitor than SKF-96365 (IC_50_ 60 μM). This means that a slight modification of the heterocyclic platform, particularly the position of the second nitrogen atom (*N*-3 position in imidazole for SKF-96365 and *N*-2 position for **7d**), has a direct impact on SOCE activity. The effect of the length for the Cβ-(4-methoxyphenylalkoxy) side chain appeared to be important in Figure 3C when we compared compounds **7c** and **7d**: for the same concentration of **7**, the curve obtained for compound **7d** gave higher effect on SOCE inhibition level (%). Again, the presence or absence of MeO group in *para*-position for the phenethyl-1*H*-pyrazolium skeleton of **7** and for the Cβ-phenylpropoxy side chain afforded variation of IC_50_, i.e., comparison of **7b** (IC_50_ 34 μM) and **7d** (IC_50_ 25 μM) showed that absence of MeO group in the skeleton of **7b** has a lower effect on SOCE IC_50_. On the other hand, absence of MeO group of **7c** (IC_50_ 48 μM) on the side chain led to a more important effect and the IC_50_ decreased. Similar observations were also made when a more physiological stimulation of SOCE activation was implemented (Figure 4). When B cells are stimulated with an antigen (M Immunoglobulin: IgM), B cell receptor (BCR) activation leads to an increase of Ca^2+^ concentration mainly due SOCE. However, inhibition by **7c** and **7d** compounds is quite identical and less than what was observed with SKF-96365.

Here, we have reported the racemic synthesis of pyrazole SKF-96365 analogues in four steps without substituent (CF_3_ group) on the pyrazole platform in moderate to good yields. For the (±) hydroxyl intermediate **4**, we have developed a successful approach for separation of enantiomers using the method of “half-concentration” with commercial (+)-(1*S*)- and (−)-(1*R*)-10-camphorsulfonic acid (CSA) followed by neutralization of diastereomers with MeONa in dry MeOH solution. With the pure enantiomers (−)-(1*S*)-**4b** and (+)-(1*R*)-**4b**, initial attempts to obtain the crystallized (−)-(1*S*)-**7d** and (+)-(1*R*)-**7d** after treatment of intermediate **6d** with a solution of 1 M HCl (for precipitation of hydrochloride salt **7d**). Effects of compounds **7a**–**d** on endoplasmic reticulum (ER) Ca^2+^ and SOCE were evaluated on PLP-B lymphocyte cell line, and **7d** was identified as a better SOCE inhibitor than SKF-96365. However, the inhibitory effects of **7c** and **7d** compounds on SOCE are inferior to what was observed for SKF-96365 when evaluated with BCR stimulation. This preliminary SAR study showed that the MeO group in *para*-position of the phenethyl-1*H*-pyrazolium skeleton or for the Cβ-phenylpropoxy side chain of **7** influenced the SOCE activity. These results offered possibilities to increase molecular diversity for a complete SAR study, and we are also currently exploring the potential of this synthetic methodology.

## 3. Materials and Methods

### 3.1. Chemistry Section

General Information. Preparative chromatography was realized on a Combi Flash *R_f_* 200 psi UV ref. 208K20284 (Serlabo Technologies, Entraigues-sur-la-Sorgue, France) using pre-packed column of alumina gel 60 F 254 Merck equipped with a DAD UV/Vis 200–360 nm detector. Thin-layer chromatography (TLC) was accomplished on 0.2 mm precoated plates of silica gel 60 F-254 (Merck) with appropriate eluent. Visualization was made with ultraviolet light (254 and 365 nm) or with a fluorescence indicator. Solvents were evaporated with a BUCHI rotary evaporator (New Castle, PA, USA). All reagents and solvents were purchased from Acros Fisher (Illkirch, France), Sigma-Aldrich Chimie (St. Quentin Fallavier, France), and Fluka Chimie (Paris, France) and were used without further purification. ^1^H NMR spectra were recorded on Bruker AC 300 P (300 MHz) spectrometer and ^13^C NMR spectra on Bruker AC 300 P (75 MHz) spectrometer. Chemical shifts are expressed in parts per million downfield. Data are given in the following order: δ value, multiplicity (s, singlet; d, doublet; t, triplet; q, quartet; quint: quintuplet, m, multiplet; br, broad), number of protons, coupling constants *J* is given in Hertz. The high-resolution mass spectra (HRMS) were recorded in positive mode using direct Electrospray infusion, respectively on Waters Q-TOF 2 or on Thermo Fisher Scientific Q-Exactive spectrometers at the “Centre Régional de Mesures Physiques de l’Ouest” platform (CRMPO platform, ScanMAT UMS 2001 CNRS, Rennes, France). Melting points were determined on a Kofler melting point apparatus and were uncorrected. Optical rotations [α_D_] were measured on a Perkin-Elmer 214 polarimeter at room temperature (25 °C) and are recorded in units of deg cm^−3^ g^−1^ dm^−1^ (*c* in g cm^−3^ in MeOH) with a 1.0 cm cell. The ee- and de-values were determined by chiral HPLC analysis using Chiracel OJ-H column (250 × 4.60 mm) with UV detector at 220 nm using hexane/*i*-PrOH as mobile phase with appropriate composition and flow rate.

*1-Phenyl-2-(1H-pyrazol-1-yl)ethan-1-one* (**3a**). To a solution of 2-bromoacetophenone **1a** (4 g, 20.1 mmol) in 20.8 mL of acetonitrile, pyrazole **2a** (1.45 g, 21.3 mmol, 1.06 equiv.) was added in small portions under vigorous magnetic stirring (550 rpm) at room temperature, and mixing was pursued until complete dissolution of the reagents. To this homogeneous solution, K_2_CO_3_ (2.92 g, 21.1 mmol, 1.05 equiv.) was poured and the resulting suspension was stirred for 8 h at 25 °C and monitored by thin layer chromatography on 0.2 mm plates of silica gel 60 F-254 (Merck) using cyclohexane/AcOEt (1:1 *v*/*v*) as eluent. The reaction mixture was diluted with 20 mL of AcOEt, and the resulting solution was filtered on a Büchner funnel (porosity N°4) and the residual precipitate was washed with AcOEt (2 × 10 mL). The collected filtrate was transferred into a separating funnel. The organic phase was washed successively with deionized water (3 × 80 mL), brine (3 × 80 mL), and dried over magnesium sulfate. After filtration on a filter paper, the filtrate was concentrated in a rotary evaporator under reduced pressure and the oily residue was submitted to purification by preparative chromatography (Combi Flash *R_f_* 200 psi apparatus with a DAD 200/360 nm detector) on pre-packed column of silica gel 60 F-254 (Merck) using a stepwise gradient of cyclohexane/AcOEt (0–50%) for elution. Pooling for 60 min and elimination of the solvent in vacuo gave 3.74 g (53% yield) of the pure desired compound **3a** as yellowish powder. Mp = 96–97 °C. ^1^H NMR (DMSO-*d*_6_) δ = 5.84 (s, 2H, CH_2_, H-2), 6.31 (dd, *J* = 2.3, 1.9 Hz, 1H, H-4′), 7.48 (dd, 1H, *J* = 1.9, 0.7 Hz, H-3′), 7.52–7.63 (m, 2H, H-3″, H-5″, Ar), 7.66–7.77 (m, 2H, H-4″, H-5′, Ar), 7.97–8.09 (m, 2H, H-2″, H-6″). ^13^C NMR (DMSO-*d*_6_) δ = 57.7 (C-2), 105.6 (C-4′), 128.1 (C-3″, C-5″), 129.0 (C-2″, C-6″), 131.6 (C-5′), 134.0 (C-4″), 134.6 (C-1″), 139.0 (C-3′), 193.7 (C=O). ES^+^ HRMS, *m*/*z* = 209.0689 found (calculated for C_11_H_10_N_2_ONa [M + Na]^+^ requires 209.0691).

*1-(4-Methoxyphenyl)-2-(1H-pyrazol-1-yl)ethan-1-one* (**3b**). To a solution of 2-bromo-1-(4-methoxyphenyl)ethan-1-one **1b** (4.6 g, 20.1 mmol) in 20.8 mL of acetonitrile, pyrazole **2a** (2.74 g, 40.2 mmol, 2 equiv.) was added in small portions under vigorous magnetic stirring (550 rpm) at room temperature, and mixing was pursued until complete dissolution of the reagents. To this homogeneous solution, K_2_CO_3_ (5.56 g, 40.2 mmol, 2 equiv.) was poured and the resulting suspension was stirred for 12 h at 25 °C and monitored by thin layer chromatography on 0.2 mm plates of silica gel 60 F-254 (Merck) using cyclohexane/AcOEt (3:7 *v*/*v*) as eluent. The reaction mixture was diluted with 20 mL of AcOEt, and the resulting suspension was filtered on a Büchner funnel (porosity N°4) and the residual precipitate was washed with AcOEt (2 × 10 mL). The collected filtrate was transferred into a separating funnel. The organic phase was washed successively with deionized water (3 × 80 mL), brine (3 × 80 mL), and dried over magnesium sulfate. After filtration on a filter paper, the filtrate was concentrated in a rotary evaporator under reduced pressure and the oily residue was submitted to purification by preparative chromatography (Combi Flash *R_f_* 200 psi apparatus with a DAD 200/360 nm detector) on pre-packed column of silica gel 60 F-254 (Merck) using a stepwise gradient of cyclohexane/AcOEt (0–70%) for elution. Pooling for 60 min and elimination of the solvent in vacuo gave 4.35 g (66% yield) of the pure desired compound **3b** as yellowish needles. Mp = 102–103 °C. ^1^H NMR (DMSO-*d*_6_) δ = 3.86 (s, 3H, OCH_3_), 5.77 (s, 2H, CH_2_, H-2), 6.30 (t, 1H, *J* = 2.1 Hz, H-4′), 7.09 (d, 2H, *J* = 8.9 Hz, H-3″, H-5″, Ar), 7.47 (dd, 1H, *J* = 1.9, 0.7 Hz, H-3′), 7.72 (dd, 1H, *J* = 2.3, 0.7 Hz, H-5′), 8.01 (d, 2H, *J* = 8.9 Hz, H-2″, H-6″, Ar). ^13^C NMR (DMSO-*d*_6_) δ = 55.7 (OCH_3_), 57.3 (C-2), 105.5 (C-4′), 114.2 (C-3″, C-5″), 127.4 (C-5′), 130.4 (C-2″, C-6″), 131.6 (C-1″), 138.8 (C-3′), 163.6 (C-4″), 191.9 (C=O). ES^+^ HRMS, *m*/*z* = 239.0798 found (calculated for C_12_H_12_N_2_O_2_Na [M + Na]^+^ requires 239.0797).

*2-(3-Trifluoromethyl-1H-pyrazol-1-yl)-1-(4-methoxyphenyl)ethan-1-one* (**3c**). To a solution of 2-bromo-1-(4-methoxyphenyl)ethan-1-one **1b** (0.5 g, 2.18 mmol) in 2.25 mL of acetonitrile, 3-trifluoromethylpyrazole **2b** (0.89 g, 6.54 mmol, 3 equiv.) was added in small portions under vigorous magnetic stirring (550 rpm) at room temperature, and mixing was pursued until complete dissolution of the reagents. To this homogeneous solution, K_2_CO_3_ (0.905 g, 6.54 mmol, 3 equiv.) was poured and the resulting suspension was stirred for 12 h at 25 °C and monitored by thin layer chromatography on 0.2 mm plates of silica gel 60 F-254 (Merck) using cyclohexane/AcOEt (1:1 *v*/*v*) as eluent. The reaction mixture was diluted with 5 mL of AcOEt, and the resulting solution was filtered on a Büchner funnel (porosity N°4) and the residual precipitate was washed with 5 mL of AcOEt. The collected filtrate was transferred into a separating funnel. The organic phase was washed successively with deionized water (3 × 20 mL), brine (3 × 20 mL), and dried over magnesium sulfate. After filtration on a filter paper, the filtrate was concentrated in a rotary evaporator under reduced pressure and gave a solid residue. After addition of 40 mL of hexane and mixing for 4 h, the solid was filtered on a Büchner funnel (porosity N°4) then dried at 60 °C for 3 h and gave 0.62 g (68% yield) of the desired compound **3c** as white powder. Mp = 146–147 °C. ^1^H NMR (DMSO-*d*_6_) δ = 3.87 (s, 3H, OCH_3_), 5.94 (s, 2H, CH_2_, H-2), 6.78 (dd, 1H, *J* = 2.4, 0.7 Hz, H-4′, Ar), 7.12 (d, 2H, *J* = 8.9 Hz, H-3″, H-5″, Ar), 7.95 (dq, 1H, *J* = 2.1, 1.0 Hz, H-5′, Ar), 8.02 (d, 2H, *J* = 8.9 Hz, H-2″, H-6″, Ar). ^13^C NMR (DMSO-*d*_6_) δ = 55.8 (OCH_3_), 58.1 (C-2), 104.4 (C-4′), 104.4 (C-4′), 114.2 (C-2″, C-6″), 114.3 (C-2″, C-6″), 127.1 (C-1″), 130.4 (C-3″, C-5″), 130.5 (C-3″, C-5″), 134.2 (C-5′), 134.3 (C-5′), 163.8 (C-4″), 191.0 (C-1). ES^+^ HRMS, *m*/*z* = 307.0671 found (calculated for C_13_H_11_N_2_O_2_F_3_Na [M + Na]^+^ requires 307.0670); 285.0862 found (calculated for C_13_H_12_N_2_O_2_F_3_ [M + H]^+^ requires 285.0851).

*2-(3,5-Bis-trifluoromethyl-1H-pyrazol-1-yl)-1-(4-methoxyphenyl)ethan-1-one* (**3d**). To a solution of 2-bromo-1-(4-methoxyphenyl)ethan-1-one **1b** (0.5 g, 2.18 mmol) in 2.25 mL of acetonitrile, 3,5-*bis*-trifluoromethylpyrazole **2c** (1.778 g, 8.72 mmol, 4 equiv.) was added in small portions under vigorous magnetic stirring (550 rpm) at room temperature, and mixing was pursued until complete dissolution of the reagents. To this homogeneous solution, K_2_CO_3_ (1.205 g, 8.72 mmol, 4 equiv.) was poured and the resulting suspension was stirred for 24 h at 25 °C and monitored by thin layer chromatography on 0.2 mm plates of silica gel 60 F-254 (Merck) using cyclohexane/AcOEt (1:1 *v*/*v*) as eluent. The reaction mixture was diluted with 5 mL of AcOEt, and the resulting solution was filtered on a Büchner funnel (porosity N°4) and the residual precipitate was washed with 5 mL of AcOEt. The collected filtrate was transferred into a separating funnel. The organic phase was washed successively with deionized water (3 × 20 mL), brine (3 × 20 mL), and dried over magnesium sulfate. After filtration on a filter paper, the filtrate was concentrated in a rotary evaporator under reduced pressure and gave a solid residue which was dried under high vacuum (10^−2^ Torr) at 25 °C for 2 h. The desired compound **3d** was obtained in 69% yield as white powder. Mp = 114–115 °C. ^1^H NMR (DMSO-*d*_6_) δ = 3.88 (s, 3H, OCH_3_), 6.19 (s, 2H, CH_2_, H-2), 7.12 (d, 2H, *J* = 8.9 Hz, H-3″, H-5″, Ar), 7.69 (s, 1H, H-4′, Ar), 8.05 (d, 1H, *J* = 8.9 Hz, H-2″, H-6″, Ar). ^13^C NMR (DMSO-*d*_6_) δ = 55.8 (OCH_3_), 58.5 (C-2), 107.5 (C-4′), 114.3 (C-3″, C-5″), 126.3 (C-1″), 130.8 (C-2″, C-6″), 164.2 (C-4″), 189.9 (C=O). ES^+^ HRMS, *m*/*z* = 375.0544 found (calculated for C_14_H_10_N_2_O_2_F_6_Na [M + Na]^+^ requires 375.0544); 353.0734 found (calculated for C_14_H_11_N_2_O_2_F_3_ [M + H]^+^ requires 353.0734).

#### 3.1.1. General Procedure for Reduction of 1-Phenyl-2-(1*H*-pyrazol-1-yl)ethan-1-one (**3a**–**c**) into 1-Phenyl-2-(1*H*-pyrazol-1-yl)ethan-1-ol (**4a**–**c**)

To a solution of 1-phenyl-2-(1*H*-pyrazol-1-yl)ethan-1-one **3** (5 mmol) in an appropriate volume of anhydrous methanol (9–40 mL), sodium borohydride NaBH_4_ (0.189 g, 5 mmol, 1 equiv.) was added in small portions at 0 °C (ice bath) under magnetic stirring (300 rpm). The resulting mixture was stirred (500 rpm) for an appropriate reaction time (5–7 h) at room temperature, and the reaction solution was monitored by thin layer chromatography (TLC) on 0.2 mm plates of silica gel 60 F-254 (Merck) using an appropriate mixture of solvents as eluent. The reaction mixture was concentrated in a rotary evaporator under reduced pressure and the resulting solid residue was washed with deionized water (3 × 45–3 × 47 mL) on a Büchner funnel. Then, the desired compounds **3a**–**c** were dried at 60 °C for 3 h.

*1-Phenyl-2-(1H-pyrazol-1-yl)ethan-1-ol* (**4a**). According to the standard procedure, the compound **4a** was prepared from 1-phenyl-2-(1*H*-pyrazol-1-yl)ethan-1-one **3a** (0.93 g, 5 mmol) in 9.3 mL of anhydrous methanol after a reaction time of 5 h using CH_2_Cl_2_/MeOH 9:1 *v*/*v* as eluent for thin layer chromatography. Washing work-up was realized with 3 × 45 mL of deionized water and gave 0.94 g (82% yield) of the desired compound **4a** as white powder. Mp = 132–134 °C. ^1^H NMR (DMSO-*d*_6_) δ = 4.14–4.30 (m, 2H, CH_2_, H-2), 4.83–5.01 (m, 1H, CH, H-1), 5.65 (br d, 1H, *J* = 4.7 Hz, OH), 6.18 (t, 1H, *J* = 2.0 Hz, H-4′), 7.19–7.39 (m, 5H, H-2″, H-3″, H-4″, H-5″, H-6″, Ar), 7.43 (dd, 1H, *J* = 1.9, 0.7 Hz, H-3′), 7.58 (dd, 1H, *J* = 2.3, 0.7 Hz, H-5′). ^13^C NMR (DMSO-*d*_6_) δ = 58.7 (C-2), 71.8 (C-1), 104.7 (C-4′), 126.1 (C-3″, C-5″), 127.4 (C-4″), 128.2 (C-2″, C-6″), 130.7 (C-5′), 138.5 (C-1″), 142.8 (C-3′). ES^+^ HRMS, *m*/*z* = 211.0848 found (calculated for C_11_H_12_N_2_O_2_Na [M + Na]^+^ requires 211.0847).

*1-(4-Methoxyphenyl)-2-(1H-pyrazol-1-yl)ethan-1-ol* (**4b**). According to the standard procedure, the compound **4b** was prepared from 1-(4-methoxyphenyl)-2-(1*H*-pyrazol-1-yl)ethan-1-one **3b** (1.08 g, 5 mmol) in 14.4 mL of anhydrous methanol after a reaction time of 7 h using CH_2_Cl_2_/MeOH 8:2 *v*/*v* as eluent for thin layer chromatography. Washing work-up was realized with 3 × 47 mL of deionized water and gave 1.091 g (86% yield) of the desired compound **4b** as white crystals with de = 50% (hexane/*i*-PrOH 96:4 *v*/*v* as eluent, flow rate = 0.6 mL/min.), retention times *t_R_*(1) = 65.92 and *t_R_*(2) = 71.38 min. [α_D_] = 0.0 (*c* 1.0, MeOH). Mp = 116–118 °C. ^1^H NMR (DMSO-*d*_6_) δ = 3.73 (s, 3H, OCH_3_), 4.10–4.27 (m, 2H, CH_2_, H-2), 4.87 (dt, 1H, *J* = 7.5, 5.1 Hz, CH, H-1), 5.54 (br d, 1H, *J* = 4.6 Hz, OH), 6.17 (dd, 1H, *J* = 1.8 Hz, H-4′), 6.88 (d, 2H, *J* = 8.7 Hz, H-3″, H-5″, Ar), 7.15–7.29 (m, 2H, H-2″, H-6″, Ar), 7.42 (dd, 1H, *J* = 1.8, 0.7 Hz, H-3′), 7.57 (dd, 1H, *J* = 2.2, 0.8 Hz, H-5′). ^13^C NMR (DMSO-*d*_6_) δ = 55.0 (OCH_3_), 58.7 (C-2), 71.4 (C-1), 104.6 (C-4′), 113.5 (C-3″, C-5″), 127.2 (C-2″, C-6″), 130.5 (C-1″), 134.8 (C-5′), 138.4 (C-3′), 158.5 (C-4″). ES^+^ HRMS, *m*/*z* = 241.0954 found (calculated for C_12_H_14_N_2_O_2_Na [M + Na]^+^ requires 241.0953).

*1-(4-Methoxyphenyl)-2-(3-trifluoromethyl-1H-pyrazol-1-yl)ethan-1-ol* (**4c**). According to the standard procedure, the compound **4c** was prepared from 1-(4-methoxyphenyl)-2-(3-trifluoromethyl-1*H*-pyrazol-1-yl)ethan-1-one **3c** (1.42 g, 5 mmol) in 39 mL of anhydrous methanol after a reaction time of 7 h using hexane/AcOEt 1:1 *v*/*v* as eluent for thin layer chromatography. Washing work-up was realized with 3 × 47 mL of deionized water and gave 1.431 g (94% yield) of the desired compound **4c** as white powder. Mp = 110–112 °C. ^1^H NMR (DMSO-*d*_6_) δ = 3.74 (s, 3H, OCH_3_), 4.27 (d, 2H, *J* = 6.5 Hz, CH_2_, H-2), 4.90 (t, 1H, *J* = 6.5 Hz, CH, H-1), 5.63 (br s, 1H, OH), 6.66 (d, 1H, *J* = 2.3 Hz, H-4′, Ar), 6.90 (d, 2H, *J* = 8.6 Hz, H-3″, H-5″, Ar), 7.28 (d, 2H, *J* = 8.3 Hz, H-2″, H-6″, Ar), 7.85 (s, 1H, H-5′, Ar). ^13^C NMR (DMSO-*d*_6_) δ = 55.0 (OCH_3_), 55.1 (OCH3), 59.3 (C-2), 71.0 (C-1), 103.7, (C-4′), 103.7 (C-4′), 113.6 (C-2″, C-6″), 113.6 (C-2″, C-6″), 127.2 (C-3″, C-5″), 127.3 (C-3″, C-5″), 133.1 (C-5′), 133.2 (C-5′), 134.2 (C-1″), 140.13 (q, *J* = 37.1 Hz, CF_3_), 158.7 (C-4″). ES^+^ HRMS, *m*/*z* = 309.0824 found (calculated for C_13_H_12_N_2_O_2_F_3_Na [M + Na]^+^ requires 309.0827); 269.0902 found (calculated for C_13_H_11_N_2_OF_3_ [M − H_2_O + H]^+^ requires 309.0827).

*2-(3,5-Bis-trifluoromethyl-1H-pyrazol-1-yl)-1-(4-methoxyphenyl)ethan-1-ol* (**4d**). To a solution of 2-(3,5-*bis*-trifluoromethyl-1*H*-pyrazol-1-yl)-1-(4-methoxyphenyl)ethan-1-one **3d** (1.76 g, 5 mmol) in 31 mL of anhydrous methanol, sodium borohydride NaBH_4_ (0.378 g, 10 mmol, 2 equiv.) was added in small portions at 0 °C (ice bath) under magnetic stirring (300 rpm). The resulting mixture was stirred (500 rpm) for 7 h at room temperature and the reaction solution was monitored by thin layer chromatography on 0.2 mm plates of silica gel 60 F-254 (Merck) using hexane/AcOEt 1:1 *v*/*v* as eluent. After concentration of the reaction mixture in vacuo, 44 mL of deionized water was added in the crude oily residue, and extraction was conducted with AcOEt (3 × 44 mL) in a separating funnel. The combined extracts were washed with 44 mL of brine and dried over magnesium sulfate. After filtration on a filter paper, the filtrate was concentrated in a rotary evaporator under reduced pressure and gave an oily residue which was dried under high vacuum (10^−2^ Torr) at 25 °C for 2 h. The desired compound **4d** (1.771 g) was obtained as yellowish mobile oil in 98% yield. ^1^H NMR (DMSO-*d*_6_) δ = 3.74 (s, 3H, OCH_3_), 4.29 (dd, 1H, *J* = 13.9, 8.9 Hz, CH, H-2), 4.40 (dd, 1H, *J* = 13.9, 4.4 Hz, CH, H-2), 5.01 (dt, 1H, *J* = 8.9, 4.4 Hz, CH, H-1), 5.73 (dd, 1H, *J* = 4.6, 0.7 Hz, OH), 6.92 (d, 2H, *J* = 8.7 Hz, H-3″, H-5″, Ar), 7.28 (d, 2H, *J* = 8.4 Hz, H-2″, H-6″, Ar), 7.50–7.54 (m, 1H, H-4′, Ar). ^13^C NMR (DMSO-*d*_6_) δ = 55.1 (OCH3), 58.8 (C-2), 70.6 (C-1), 106.8 (C-4′), 113.7 (C-3″, C-5″), 127.2 (C-2″, C-6″), 133.5 (C-1″), 158.9 (C-4″). ES^+^ HRMS, *m*/*z* = 377.0700 found (calculated for C_14_H_12_N_2_O_2_F_6_Na [M + Na]^+^ requires 377.0701); 337.0763 found (calculated for C_14_H_11_N_2_OF_6_ [M − H_2_O + H]^+^ requires 337.0776).

#### 3.1.2. General Procedure for the Synthesis of (1*R*, 1*S*) 1-[β-(Phenylalkoxy)-phenethyl]-1*H*-pyrazolium Hydrochloride (**7a**–**e**)

Pellets of potassium hydroxide KOH (0.112 g, 2 mmol, 2 equiv.) were added to a solution of 1-phenyl-2-(1*H*-pyrazol-1-yl)ethan-1-ol **4** (1 mmol, 1 equiv.) in 3.76 mL of dry dimethylsulfoxide pa at room temperature. The resulting mixture was stirred vigorously (550 rpm) for 30 min until complete dissolution of KOH, then commercial arylalkyl halide **5** (1 mmol, 1 equiv.) was added in one portion in the reaction mixture. The reaction mixture was heated at 50 °C under magnetic stirring (300 rpm) for 48 h. After cooling down to room temperature, 18.7 mL of saturated brine was added to the reaction mixture and the resulting solution was transferred into a separating funnel. Extraction was conducted with 18.7 mL of diethyl ether Et_2_O, then the organic phase was dried over magnesium sulphate and filtered on a filter paper. The filtrate was concentrated in a rotary evaporator under reduced pressure. The oily crude residue containing1-[β-(phenylalkoxy)-phenethyl]-1*H*-pyrazole **6** was dissolved in appropriate volume of diethyl ether Et_2_O pa under a stream of argon with magnetic stirring (200 rpm). To this homogeneous solution, a commercial solution of 1 M HCl (1 equiv.) in ether was added dropwise rapidly. During mixing at room temperature, the initial pale yellow oil crystallized progressively on the circumference of the round flask and mixing (from 24 h to 5 days) was pursued until complete crystallization of the desired 1-[β-(phenylalkoxy)-phenethyl]-1*H*-pyrazolium hydrochloride **7**. The resulting precipitate was collected by filtration on a Büchner funnel (porosity N°4) and washed with dry diethyl ether pa. The desired compound **7** was dried at room temperature for 4 h and stored in a dessicator.

*(1R, 1S) 1-[β-((4-Methoxybenzyl)oxy)-phenethyl]-1H-pyrazolium hydrochloride* (**7a**). Using the standard procedure from 1-phenyl-2-(1*H*-pyrazol-1-yl)ethan-1-ol **4a** (0.188 g, 1 mmol) and 4-methoxybenzyl chloride **5a** (0.157 g, 1 mmol, 1 equiv.), the 1-[β-((4-methoxybenzyl)oxy)-phenethyl]-1*H*-pyrazole **6a** (0.199 g, 0.65 mmol) was dissolved in 4.6 mL of dry diethyl ether pa under a stream of argon. The addition of 0.65 mL of 1M HCl (0.65 mmol, 1 equiv.) produced a viscous gum on the circumference of the round flask after mixing at room temperature for 5 days. Then, this reaction mixture was stored in a refrigerator at 4 °C for 2 weeks and produced a compact gum. This gum was then manually and carefully triturated in 4 mL of dry hexane and progressively produced a powder. The resulting suspension was stirred (200 rpm) at room temperature for 5 days, and produced a completely divided powder. The desired salt **7a** was collected by filtration on a Büchner funnel (porosity N°4) and washed with 0.5 mL of dry diethyl ether pa. After drying at 25 °C for 4 h, the 1-[β-((4-methoxybenzyl)oxy)-phenethyl]-1*H*-pyrazolium hydrochloride **7a** (35.72 mg, 10% yield) was obtained as white powder. Mp = 62–65 °C. ^1^H NMR (DMSO-*d*_6_) δ = 3.72 (s, 3H, OCH_3_), 4.09 (d, 1H, *J* = 11.6 Hz, CH, H-1″), 4.19–4.32 (m, 2H, CH, H-1″, H-1′), 4.40 (dd, 1H, *J* = 13.9, 8.5 Hz, CH, H-1′), 4.78 (dd, 1H, *J* = 8.4, 4.4 Hz, CH, H-2′), 6.22 (t, 1H, *J* = 2.0 Hz, H-4, Ar), 6.82 (d, 2H, *J* = 8.6 Hz, H-3b, H-5b, Ar), 6.99 (d, 2H, *J* = 8.6 Hz, H-2b, H-6b, Ar), 7.27–7.44 (m, 5H, H-2a, H-3a, H-4a, H-5a, H-6a), 7.46 (d, 1H, *J* = 1.8 Hz, H-3, Ar), 7.62 (d, 1H, *J* = 2.2 Hz, H-5, Ar). ^13^C NMR (DMSO-*d*_6_) δ = 55.1 (OCH_3_), 57.0 (C-1′), 69.7 (C-1″), 79.4 (C-2′), 104.9 (C-4), 113.6 (C-3a, C-5a), 126.8 (C-2a, C-6a), 128.1 (C-4a), 128.6 (C-2a, C-6a), 128.8 (C-3a, C-5a), 129.9 (C-1b), 130.9 (C-5), 138.6 (C-3), 139.0 (C-1a), 158.6 (C-4b). ES^+^ HRMS, *m*/*z* = 331.1418 found (calculated for C_19_H_20_N_2_O_2_Na [M + Na]^+^ requires 331.1417).

*(1R, 1S) 1-[β-((3-(4-Methoxyphenyl)propyl)oxy)-phenethyl]-1H-pyrazolium hydrochloride* (**7b**). Using the standard procedure from 1-phenyl-2-(1*H*-pyrazol-1-yl)ethan-1-ol **4a** (0.188 g, 1 mmol) and 1-(3-bromopropyl)-4-methoxybenzene **5c** (0.229 g, 1 mmol, 1 equiv.), the 1-[β-((3-phenylpropyl)oxy)-phenethyl]-1*H*-pyrazole **6b** (0.151 g, 0.45 mmol) was dissolved in 3 mL of dry diethyl ether pa under a stream of argon. The addition of 0.45 mL of 1M HCl (0.45 mmol, 1 equiv.) produced a pale yellow viscous oil that crystallized progressively on the circumference of the round flask, and mixing (200 rpm, 72 h) was pursued until complete crystallization of the oily gum. The desired salt **7b** was collected by filtration on a Büchner funnel (porosity N°4) and washed with 3 × 0.5 mL of dry diethyl ether pa. After drying at 25 °C for 4 h, the 1-[β-((3-(4-methoxyphenyl)propyl)oxy)-phenethyl]-1*H*-pyrazolium hydrochloride **7b** (78.3 mg, 21% yield) was obtained as white powder. Mp = 76–80 °C. ^1^H NMR (DMSO-*d*_6_) δ = 1.62 (dt, 2H, *J* = 8.2, 6.2 Hz, CH_2_, H-2″), 2.36 (dd, 2H, *J* = 8.4, 6.7 Hz, CH_2_, H-3″), 3.05 (dt, 1H, *J* = 9.5, 6.1 Hz, CH, H-1″), 3.22 (dt, 1H, *J* = 9.5, 6.0 Hz, CH, H-1″), 3.69 (s, OCH_3_), 4.23 (dd, 1H, *J* = 13.9, 4.4 Hz, CH, H-1′), 4.37 (dd, 1H, *J* = 13.9, 8.7 Hz, CH, H-1′), 4.69 (dd, 1H, *J* = 8.7, 4.2 Hz, CH, H-2′), 6.23 (t, 1H, *J* = 2.1 Hz, H-4, Ar), 6.77 (d, 2H, *J* = 8.6 Hz, H-3b, H-5b, Ar), 6.94 (d, 2H, *J* = 8.6 Hz, H-2b, H-6b, Ar), 7.27–7.44 (m, 5H, H-2a, H-3a, H-4a, H-5a, H-6a, Ar), 7.46–7.50 (m, 1H, H-3), 7.68 (dd, 1H, *J* = 2.3, 0.7 Hz, H-5). ^13^C NMR (DMSO-*d*_6_) δ = 30.4 (C-3″), 31.1 (C-2″), 54.9 (OCH3), 57.2 (C-1′), 67.4 (C-1″), 80.1 (C-2′), 104.9 (C-4), 113.6 (C-3b, C-5b), 126.7 (C-2a, C-6a), 128.0 (C-4a), 128.5 (C-3a, C-5a), 129.2 (C-2b, C-6b), 131.0 (C-5), 133.4 (C-1b), 138.4 (C-3), 139.3 (C-1a), 157.3 (C-4b). ES^+^ HRMS, *m*/*z* = 359.1733 found (calculated for C_21_H_24_N_2_O_2_Na [M + Na]^+^ requires 359.1735); 337.1906 found (calculated for C_21_H_25_N_2_O_2_ [M + H]^+^ requires 337.1916).

*(1R, 1S) 1-[β-((4-Methoxybenzyl)oxy)-(4-methoxyphenethyl]-1H-pyrazolium hydrochloride* (**7c**). Using the standard procedure from 1-(4-methoxyphenyl)-2-(1*H*-pyrazol-1-yl)ethan-1-ol **4b** (0.218 g, 1 mmol) and 4-methoxybenzyl chloride **5a** (0.157 g, 1 mmol, 1 equiv.), the 1-[β-((4-methoxybenzyl)oxy)-(4-methoxyphenethyl]-1*H*-pyrazole **6c** (0.231 g, 0.68 mmol) was dissolved in 4.57 mL of dry diethyl ether pa under a stream of argon. The addition of 0.68 mL of 1M HCl (0.68 mmol, 1 equiv.) produced a pale pink viscous oil that crystallized rapidly (30 min.) on the circumference of the round flask, and mixing (200 rpm, 72 h) was pursued until complete crystallization of the viscous gum into divided powder. The desired salt **7c** was collected by filtration on a Büchner funnel (porosity N°4) and washed with 3 × 0.5 mL of dry diethyl ether pa. After drying at 25 °C for 4 h, the 1-[β-((4-methoxybenzyl)oxy)-(4-methoxyphenethyl]-1*H*-pyrazolium hydrochloride **7c** (123.7 mg, 33% yield) was obtained as white powder. Mp = 112–113 °C. ^1^H NMR (DMSO-*d*_6_) δ = 3.72 (s, 3H, OCH_3_), 3.76 (s, 3H, OCH_3_), 4.06 (d, 1H, *J* = 11.6 Hz, CH, H-1″), 4.16–4.30 (m, 2H, CH, H-1′, H-1″), 4.40 (dd, 1H, *J* = 13.8, 8.5 Hz, CH, H-1′), 4.71 (dd, 1H, *J* = 8.4, 4.5 Hz, CH, H-2′), 6.22 (t, 1H, *J* = 2.0 Hz, H-4, Ar), 6.82 (d, 2H, *J* = 8.6 Hz, H-3a, H-5a, Ar), 6.89–7.03 (m, 4H, H-2a H-6a, H-3b, H-5b, Ar), 7.26 (d, 2H, *J* = 8.6 Hz, H-2b, H-6b, Ar), 7.47 (d, 1H, *J* = 1.9 Hz, H-3, Ar), 7.62 (d, 1H, *J* = 2.2 Hz, H-5, Ar). ^13^C NMR (DMSO-*d*_6_) δ = 55.1 (OCH_3_), 55.1 (OCH_3_), 57.0 (C-1′), 69.3 (C-1″), 78.9 (C-2′), 104.9 (C-4), 113.6 (C-3a, C-5a), 114.0 (C-3b, C-5b), 128.2 (C-2a C-6a), 128.8 (C-2b, C-6b), 130.0 5 (C-1a), 130.8 (C-1b), 130.9 (C-5), 138.5 (C-3), 158.6 (C-4a), 159.1 (C-4b). ES^+^ HRMS, *m*/*z* = 361.1526 found (calculated for C_20_H_22_N_2_O_3_Na [M + Na]^+^ requires 361.1528); 339.1715 found (calculated for C_20_H_23_N_2_O_3_ [M + H]^+^ requires 339.1709). 

*(1R, 1S) 1-[β-((4-Methoxyphenyl)-(3-(4-methoxyphenyl)propoxyethyl)]-1H-pyrazolium hydrochloride* (**7d**). Using the standard procedure from 1-(4-methoxyphenyl)-2-(1*H*-pyrazol-1-yl)ethan-1-ol **4b** (0.218 g, 1 mmol) and 1-(3-bromopropyl)-4-methoxybenzene **5c** (0.229 g, 1 mmol, 1 equiv.), the 1-[β-((4-methoxyphenyl)-(3-(4-methoxyphenyl)propoxyethyl)]-1*H*-pyrazole **6d** (0.156 g, 0.42 mmol) was dissolved in 2.84 mL of dry diethyl ether pa under a stream of argon. The addition of 0.42 mL of 1M HCl (0.42 mmol, 1 equiv.) produced a pale pink viscous oil that crystallized (12 h) on the circumference of the round flask, and mixing (200 rpm, 72 h) was pursued until complete crystallization of the viscous gum. The desired salt **7d** was collected by filtration on a Büchner funnel (porosity N°4) and washed with 3 × 0.5 mL of dry diethyl ether pa. After drying at 25 °C for 4 h, the 1-[β-((4-methoxyphenyl)-(3-(4-methoxyphenyl)propoxyethyl)]-1*H*-pyrazolium hydrochloride **7d** (72.53 mg, 18% yield) was obtained as white powder. Mp = 92–94 °C. ^1^H NMR (DMSO-*d*_6_) δ = 1.47–1.70 (m, 2H, CH_2_, H-2″), 2.36 (t, 2H, *J* = 7.5 Hz, CH_2_, H-3″), 3.03 (dt, 1H, *J* = 9.6, 6.2 Hz, CH, H-1″), 3.19 (dt, 1H, *J* = 9.5, 6.0 Hz, CH, H-1″), 3.69 (s, 3H, OCH_3_), 3.75 (s, 3H, OCH_3_), 4.18 (dd, 1H, *J* = 13.8, 4.3 Hz, CH, H-1′), 4.35 (dd, 1H, *J* = 13.8, 8.7 Hz, CH, H-1′), 4.62 (dd, 1H, *J* = 8.7, 4.2 Hz, CH, H-2′), 6.22 (t, 1H, *J* = 2.0 Hz, H-4, Ar), 6.77 (d, 2H, *J* = 8.6 Hz, H-3b, H-5b, Ar), 6.86–7.02 (m, 4H, H-3a, H-5a, H-2b, H-6b, Ar), 7.26 (d, 2H, *J* = 8.6 Hz, H-2a, H-6a, Ar), 7.47 (d, 1H, *J* = 1.8 Hz, H-3, Ar), 7.67 (d, 1H, *J* = 2.2 Hz, H-5, Ar). ^13^C NMR (DMSO-*d*_6_) δ = 30.4 (C-3″), 31.1 (C-2″), 54.9 (OCH_3_), 55.1 (OCH_3_), 57.2 (C-1′), 67.0 (C-1″), 79.7 (C-2′), 104.9 (C-4), 113.6 (C-3b, C-5b), 113.9 (C-3a, C-5a), 128.0 (C-2a, C-6a), 129.2 (C-2b, C-6b), 130.8 (C-3), 131.1 (C-1a), 133.4 (C-1b), 138.5 (C-5), 157.3 (C-4b), 159.0 (C-4a). ES^+^ HRMS, *m*/*z* = 389.1838 found (calculated for C_22_H_26_N_2_O_3_Na [M + Na]^+^ requires 389.1841).

*(1R, 1S) 1-[β-((4-Methoxyphenyl)-(3-phenylpropoxyethyl)]-1H-pyrazolium hydrochloride* (**7e**). Using the standard procedure from 1-(4-methoxyphenyl)-2-(1*H*-pyrazol-1-yl)ethan-1-ol **4b** (0.218 g, 1 mmol) and 1-bromo-3-phenylpropane **5d** (0.199 g, 1 mmol, 1 equiv.), the 1-[β-((4-methoxyphenyl)-(3-phenylpropoxyethyl)]-1*H*-pyrazole **6e** (0.124 g, 0.37 mmol) was dissolved in 2.5 mL of dry diethyl ether pa under a stream of argon. The addition of 0.37 mL of 1 M HCl (0.37 mmol, 1 equiv.) produced a translucent colourless viscous oil that crystallized progressively (24 h) on the circumference of the round flask, and mixing (200 rpm, 72 h) was pursued until complete crystallization in divided powder. The desired salt **7e** was collected by filtration on a Büchner funnel (porosity N°4) and washed with 3 × 0.5 mL of dry diethyl ether pa. After drying at 25 °C for 4 h, the 1-[β-((4-methoxyphenyl)-(3-phenylpropoxyethyl)]-1*H*-pyrazolium hydrochloride **7d** (82.04 mg, 22% yield) was obtained as white powder. Mp = 87–89 °C. ^1^H NMR (DMSO-*d*_6_) δ = 1.54–1.76 (m, 2H, CH_2_, H-2″), 2.37–2.46 (m, 2H, CH_2_, H-3″), 3.04 (dt, 1H, *J* = 9.6, 6.1 Hz, CH, H-1″), 3.21 (dt, 1H, *J* = 9.6, 6.0 Hz, CH, H-1″), 3.75 (s, 3H, OCH_3_), 4.18 (dd, 1H, *J* = 13.8, 4.3 Hz, CH, H-1′), 4.35 (dd, 1H, *J* = 13.8, 8.7 Hz, CH, H-1′), 4.63 (dd, 1H, *J* = 8.7, 4.3 Hz, CH, H-2′), 6.21 (t, 1H, *J* = 2.1 Hz, H-4, Ar), 6.92 (d, 2H, *J* = 8.7 Hz, H-3a, H-5a, Ar), 6.99–7.06 (m, 2H, H-2b, H-6b, Ar), 7.08–7.17 (m, 1H, H-4b, Ar), 7.17–7.31 (m, 4H, H-2a H-6a, H-3b, H-5b, Ar), 7.46 (dd, 1H, *J* = 1.9, 0.7 Hz, H-3, Ar), 7.66 (dd, 1H, *J* = 2.3, 0.7 Hz, H-5, Ar). ^13^C NMR (DMSO-*d*_6_) δ = 30.9 (C-2″), 31.4 (C-3″), 55.1 (OCH_3_), 57.2 (C-1′), 67.1 (C-1″), 79.7 (C-2′), 104.9 (C-4), 113.9 (C-3a, C-5a), 125.7 (C-4b), 128.1 (C-2a, C-6a), 128.2 (C-3b, C-5b), 128.4 (C-2b, C-6b), 130.8 (C-5), 131.2 (C-1a), 138.6 (C-3), 141.7 (C-1b), 159.1 (C-4a). ES^+^ HRMS, *m*/*z* = 359.1736 found (calculated for C_21_H_24_N_2_O_2_Na [M + Na]^+^ requires 359.1736).

#### 3.1.3. Resolution of (±)-(1*R*, 1*S*) 1-(4-Methoxyphenyl)-2-(1*H*-pyrazol-1-yl)ethan-1-ol (**4b**) with (−)-(1R)-10-Camphorsulfonic Acid and (+)-(1S)-10-Camphorsulfonic Acid by Two Convergent Methods of Half-Quantities

##### Method 1 from (−)-(1R)-CSA:

*(−)-(1S) 1-(4-methoxyphenyl)-2-(1H-pyrazol-1-yl)ethan-1-ol* (**4b**): To a stirred solution of (±) 1-(4-methoxyphenyl)-2-(1*H*-pyrazol-1-yl)ethan-1-ol **4b** (0.5 g, 2.29 mmol) in 5 mL of dry acetone, a solution of (−)-(1*R*)-10-camphorsulfonic acid (0.266 g, 1.145 mmol) in 2 mL of dry acetone was added dropwise at room temperature for 5 min. Stirring (300 rpm) was pursued for 12 h at 25 °C. It is interesting to note that during the addition of the solution of (−)-(1*R*)-CSA, the formation of a white fine suspension of diastereomer appeared in the reaction mixture. This first fraction of diastereomer salt was collected by filtration on a Büchner funnel (porosity N°4), washed thoroughly with 4 × 0.3 mL of dry acetone, and dried in vacuum to give 0.363 g of the desired the salt (−)-(1*S*)-**4b**/(−)-(1*R*)-CSA (>85% de). This salt was recrystallized in dry acetone and afforded 0.271 g (52% isolated yield) of salt (−)-(1*S*)-**4b**/(−)-(1*R*)-CSA (>97% de). This salt was dissolved in 0.6 mL of dry acetone under magnetic stirring (300 rpm), and to this suspension, 0.02 equiv. of racemic (±) 1-(4-methoxyphenyl)-2-(1*H*-pyrazol-1-yl)ethan-1-ol **4b** was added for diastereomeric enrichment. This suspension was stirred for 18 h at room temperature. The enriched salt was successively filtered on a Büchner funnel, washed with 0.5 mL of dry acetone, dried in vacuum, and recrystallized in dry acetone that produced 0.251 g (48% yield) of (−)-(1*S*)-**4b**/(−)-(1*R*)-CSA (>99% de) as white powder; [α_D_] = −6,0 (*c* 1.0, MeOH).

Starting from 200 mg of the pure salt (−)-(1*S*)-**4b**/(−)-(1*R*)-CSA, a mixed suspension was prepared in 2.5 mL of dry methanol and 1 equiv. of commercial MeONa was added in one portion. The resulting reaction mixture was stirred at room temperature for 12 h and then concentrated in a rotary evaporator under reduced pressure. The crude solid residue was washed thoroughly with 4 × 2 mL of deionized water. The insoluble compound was collected by filtration on a Büchner funnel (porosity N°4) and dried in vacuum, which produced 0.096 g (38% yield) of the desired (−)-(1*S*) 1-(4-methoxyphenyl)-2-(1*H*-pyrazol-1-yl)ethan-1-ol **4b** (99% ee); [α_D_] = −10,0 (*c* 1.0, MeOH) and Mp = 113–114 °C.

*Recovery of the (+)-(1R) 1-(4-methoxyphenyl)-2-(1H-pyrazol-1-yl)ethan-1-ol* **4b**: The filtrate of the first fraction was submitted to concentration in a rotary evaporator under reduced pressure and afforded 0.345 g of a crude residue. To this solid residue, 14.5 mL of dry toluene was added and the resulting suspension was submitted to magnetic stirring for 4 h. This suspension was filtered through a Büchner funnel (porosity N°4) and the filtrate was concentrated in a rotary evaporator under reduced pressure and produced 0.190 g (0.87 mmol) of a white powder of (+)-(1*R*)-**4b** (65% ee). This white solid was dissolved in 3.8 mL of dry acetone under magnetic stirring and to the resulting solution, a solution of (+)-(1*S*)-CSA (0.2 g, 0.87 mmol, 1 equiv.) in 1.52 mL of dry acetone was added dropwise. During the addition of the (+)-(1*S*)-CSA solution, a fine suspension appeared in the mixture, which was stirred at room temperature for 12 h. The new diastereomer salt was collected by filtration then washed with 4 × 0.25 mL of dry acetone, dried in vacuum, which gave 0.249 g (63% yield) of the (+)-(1*R*)-**4b**/(+)-(1*S*)-CSA salt (96% de) as white powder. From this salt, diastereomeric enrichment was conducted with 0.04 equiv. of racemic (±) 1-(4-methoxyphenyl)-2-(1*H*-pyrazol-1-yl)ethan-1-ol **4b**. The enriched suspension was stirred for 18 h at room temperature and the resulting enriched salt was successively submitted to filtration on a Büchner funnel, washing with 0.5 mL of dry acetone, drying in vacuum, and finally recrystallisation in dry acetone which gave 0.202 mg (51% yield) of (+)-(1*R*)-**4b**/(+)-(1*S*)-CSA (>99% de) as white needles; [α_D_] = +6,0 (*c* 1.0, MeOH).

Starting from 200 mg of the pure salt (+)-(1*R*)-**4b**/(+)-(1*S*)-CSA, a mixed suspension was prepared in 2.5 mL of dry methanol, and 1 equiv. of commercial MeONa was added in one portion. The resulting reaction mixture was stirred at room temperature for 12 h and then concentrated in a rotary evaporator under reduced pressure. The crude solid residue was washed thoroughly with 4 × 2 mL of deionized water. The insoluble compound was collected by filtration on a Büchner funnel (porosity N°4) and dried in vacuum which gave 0.078 g (41% yield) of the desired (+)-(1*R*) 1-(4-methoxyphenyl)-2-(1*H*-pyrazol-1-yl)ethan-1-ol **4b** (99% ee); [α_D_] = +10,0 (*c* 1.0, MeOH) and Mp = 110–111 °C.

##### Method 2 with (+)-(1S)-CSA:

*(+)-(1R) 1-(4-Methoxyphenyl)-2-(1H-pyrazol-1-yl)ethan-1-ol* (**4b**): To a stirred solution of (±) 1-(4-methoxyphenyl)-2-(1*H*-pyrazol-1-yl)ethan-1-ol **4b** (0.5 g, 2.29 mmol) in 5 mL of dry acetone, a solution of (+)-(1*S*)-10-camphorsulfonic acid (0.266 g, 1.145 mmol) in 2 mL of dry acetone was added dropwise at room temperature for 5 min. Stirring (300 rpm) was pursued for 12 h at 25 °C. It is interesting to note that during the addition of the solution of (+)-(1*S*)-CSA, the formation of a white fine suspension of diastereomer appeared in the reaction mixture. This first fraction of diastereomer salt was collected by filtration on a Büchner funnel (porosity N°4), washed thoroughly with 4 × 0.3 mL of dry acetone, and dried in vacuum to give 0.358 g of the desired the salt (+)-(1*R*)-**4b**/(+)-(1*S*)-CSA (>87% de). This salt was recrystallized in dry acetonitrile and afforded 0.280 g (54% isolated yield) of salt (+)-(1*R*)-**4b**/(+)-(1*S*)-CSA (>96% de). This salt was dissolved in 0.6 mL of dry acetone under magnetic stirring (300 rpm) and to this suspension was added 0.02 equiv. of racemic (±) 1-(4-methoxyphenyl)-2-(1*H*-pyrazol-1-yl)ethan-1-ol **4b** for diastereomeric enrichment. This suspension was stirred for 18 h at room temperature. The enriched salt was successively filtered on a Büchner funnel, washed with 0.5 mL of dry acetone, dried in vacuum, and recrystallized in dry acetone that gave 0.229 g (44% yield) of (+)-(1*R*)-**4b**/(+)-(1*S*)-CSA (>99% de) as white powder; [α_D_] = +6,0 (*c* 1.0, MeOH).

Starting from 200 mg of the pure salt (+)-(1*R*)-**4b**/(+)-(1*S*)-CSA, a mixed suspension was prepared in 2.5 mL of dry methanol, and 1 equiv. of commercial MeONa was added in one portion. The resulting reaction mixture was stirred at room temperature for 12 h and then concentrated in a rotary evaporator under reduced pressure. The crude solid residue was washed thoroughly with 4 × 2 mL of deionized water. The insoluble compound was collected by filtration on a Büchner funnel (porosity N°4) and dried in vacuum, which gave 0.089 g (35% yield) of the desired (+)-(1*R*) 1-(4-methoxyphenyl)-2-(1*H*-pyrazol-1-yl)ethan-1-ol **4b** (99% ee); [α_D_] = +10,0 (*c* 1.0, MeOH) and Mp = 110–111 °C.

*Recovery of the (−)-(1S) 1-(4-methoxyphenyl)-2-(1H-pyrazol-1-yl)ethan-1-ol* **4b**: The filtrate of the first fraction was submitted to concentration in a rotary evaporator under reduced pressure and afforded 0.366 g of a crude residue. To this solid residue, 14.5 mL of dry toluene was added and the resulting suspension was submitted to magnetic stirring for 4 h. This suspension was filtered through a Büchner funnel (porosity N°4) and the filtrate was concentrated in a rotary evaporator under reduced pressure and produced 0.207 g (0.95 mmol) of a white powder of (−)-(1*S*)-**4b** (74% ee). This white solid was dissolved in 4.2 mL of dry acetone under magnetic stirring and to the resulting solution, a solution of (−)-(1*R*)-CSA (0.219 g, 0.95 mmol, 1 equiv.) in 1.66 mL of dry acetone was added dropwise. During the addition of the (−)-(1*R*)-CSA solution, a fine suspension appeared in the mixture, which was stirred at room temperature for 12 h. The new diastereomer salt was collected by filtration then washed with 4 × 0.25 mL of dry acetone and dried in vacuum, which gave 0.285 g (66% yield) of the (−)-(1*S*)-**4b**/(−)-(1*R*)-CSA salt (98% de) as white powder. From this salt, diastereomeric enrichment was conducted with 0.01 equiv. of racemic (±) 1-(4-methoxyphenyl)-2-(1*H*-pyrazol-1-yl)ethan-1-ol **4b**. The enriched suspension was stirred for 18 h at room temperature and the resulting enriched salt was successively submitted to filtration on a Büchner funnel, washing with 0.5 mL of dry acetone, drying in vacuum, and finally recrystallisation in dry acetone which gave 0.258 mg (60% yield) of (−)-(1*S*)-**4b**/(−)-(1*R*)-CSA (>99% de) as white needles; [α_D_] = −6,0 (*c* 1.0, MeOH).

Starting from 200 mg of the pure salt (−)-(1*S*)-**4b**/(−)-(1*R*)-CSA, a mixed suspension was prepared in 2.5 mL of dry methanol, and 1 equiv. of commercial MeONa was added in one portion. The resulting reaction mixture was stirred at room temperature for 12 h and then concentrated in a rotary evaporator under reduced pressure. The crude solid residue was washed thoroughly with 4 × 2 mL of deionized water. The insoluble compound was collected by filtration on a Büchner funnel (porosity N°4) and dried in vacuum which gave 0.090 g (43% yield) of the desired (−)-(1*S*) 1-(4-methoxyphenyl)-2-(1*H*-pyrazol-1-yl)ethan-1-ol **4b** (>99% ee); [α_D_] = −10,0 (*c* 1.0, MeOH) and Mp = 113-114 °C.

*(−)-(1S)*-**4b***/(−)-(1R)-CSA salt*: Yield = 48%. White powder, Mp = 168–170 °C. [α_D_] = −6.0 (*c* 1.0, MeOH) and >99% de (retention time *t_R_* = 34.01 min. using hexane/*i*-PrOH 94:6 *v*/*v* as eluent, flow rate = 0.8 mL/min.). ^1^H NMR (DMSO-*d*_6_) δ = 0.75 (s, 3H, C-7″CH_3_), 1.04 (s, 3H, C-7″CH_3_), 1.19–1.40 (m, 2H, CH_2_, H-2″, H-5″), 1.81 (d, 1H, *J* = 18.1 Hz, CH, H-4″), 1.80–1.92 (m, 1H, CH, H-5″), 1.95 (t, 1H, *J* = 4.5 Hz, CH, H-4″), 2.16–2.34 (m, 1H, CH, H-3″), 2.46 (d, 1H, *J* = 14.7 Hz, CH, CH_2_SO_3_H), 2.55–2.76 (m, 1H, CH, H-2″), 2.93 (d, 1H, *J* = 14.7 Hz, CH, CH_2_SO_3_H), 3.73 (s, 3H, OCH_3_), 4.10–4.32 (m, 2H, CH_2_, H-2), 4.87 (dd, 1H, *J* = 7.3, 5.5 Hz, CH, H-1), 6.20 (t, 1H, *J* = 2.1 Hz, H-4′, Ar), 6.87 (d, 2H, *J* = 8.7 Hz, H-3a, H-5a, Ar), 7.22 (d, 2H, *J* = 8.5 Hz, H-2a, H-6a, Ar), 7.47 (dd, 1H, *J* = 1.9, 0.7 Hz, H-3′, Ar), 7.61 (dd, 1H, *J* = 2.3, 0.7 Hz, H-5′, Ar). ^13^C NMR (DMSO-*d*_6_) δ = 19.5 (C-7″CH_3_), 20.0 (C-7″CH_3_), 24.2 (C-2″), 26.4 (C-5″), 42.1 (C-3″), 42.2 (C-4″), 46.8 (CH_2_SO_3_H), 47.1 (C-7″), 55.0 (OCH3), 58.1 (C-6″), 58.6 (C-2), 71.3 (C-1), 104.8 (C-4′), 113.5 (C-3a, C-5a), 127.2 (C-2a, C-6a), 130.9 (C-5′), 134.7 (C-1a), 138.1 (C-3′), 158.5 (C-4a), 216.0 (C=O). ES^+^ HRMS, *m*/*z* = 241.0946 found (calculated for C_12_H_14_N_2_O_2_Na [M – H + Na]^+^ requires 241.0947).

*(+)-(1R)*-**4b***/(+)-(1S)-CSA salt*: Yield = 51%. White powder, Mp = 169-171 °C. [α_D_] = +6.0 (*c* 1.0, MeOH) and >99% de (retention time *t_R_* = 35.5 min. using hexane/*i*-PrOH 94:6 v/v as eluent, flow rate = 0.8 mL/min.). ^1^H NMR (DMSO-*d*_6_) δ = 0.75 (s, 3H, C-7″CH_3_), 1.04 (s, 3H, C-7″CH3), 1.17–1.42 (m, 2H, CH_2_, H-2″, H-5″), 1.81 (d, 1H, *J* = 18.1 Hz, CH, H-4″), 1.78–1.93 (m, 1H, CH, H-5″), 1.95 (t, 1H, *J* = 4.5 Hz, CH, H-4″), 2.25 (dt, 1H, *J* = 18.0, 4.0 Hz, CH, H-3″), 2.45 (d, 1H, *J* = 14.7 Hz, CH, CH_2_SO_3_H), 2.54-2.76 (m, 1H, CH, H-2″), 2.93 (d, 1H, *J* = 14.7 Hz, CH, CH_2_SO_3_H), 3.73 (s, 3H, OCH_3_), 4.08–4.32 (m, 2H, CH_2_, H-2), 4.87 (dd, 1H, *J* = 7.3, 5.5 Hz, CH, H-1), 6.20 (t, 1H, *J* = 2.1 Hz, H-4′, Ar), 6.87 (d, 2H, *J* = 8.7 Hz, H-3a, H-5a, Ar), 7.22 (d, 2H, *J* = 8.5 Hz, H-2a, H-6a, Ar), 7.43-7.41 (m, 1H, H-3′, Ar), 7.60 (dd, 1H, *J* = 2.3, 0.7 Hz, H-5′, Ar). ^13^C NMR (DMSO-*d*_6_) δ = 19.5 (C-7″CH_3_), 20.0 (C-7″CH_3_), 24.2 (C-2″), 26.4 (C-5″), 42.1 (C-3″), 42.2 (C-4″), 46.8 (CH_2_SO_3_H), 47.1 (C-7″), 55.0 (OCH_3_), 58.1 (C-6″), 58.6 (C-2), 71.3 (C-1), 104.8 (C-4′), 113.5 (C-3a, C-5a), 127.2 (C-2a, C-6a), 130.9 (C-5′), 134.7 (C-1a), 138.1 (C-3′), 158.5 (C-4a), 216.0 (C=O). ES^+^ HRMS, *m/z* = 241.0946 found (calculated for C_12_H_14_N_2_O_2_Na [M − H + Na]^+^ requires 241.0947).

X-ray crystallographic data for (+)-(1*R*)-**4b**/(+)-(1*S*)-CSA salt: (C_12_H_15_N_2_O_2_, C_10_H_15_O_4_S); *M* = 450.54. APEXII, Bruker-AXS diffractometer, Mo-Kα radiation (λ = 0.71073 Å), *T* = 150(2) K; orthorhombic *P* 21 21 21 (I.T.#19), a = 7.2781(5), b = 9.5344(5), c = 32.8134(19) Å, *V* = 2277.0(2) Å^3^. *Z* = 4, *d* = 1.314 g·cm^−3^, μ = 0.182 mm^−1^. The structure was solved by direct methods using the *SIR97* program [29], and then refined with full-matrix least-square methods based on *F*2 (*SHELXL-97*) [30] with the aid of the *WINGX* [31] program. All non-hydrogen atoms were refined with anisotropic atomic displacement parameters. Except oxygen- and nitrogen-linked hydrogen atoms that were introduced in the structural model through Fourier difference maps analysis, H atoms were finally included in their calculated positions. A final refinement on *F*^2^ with 4854 unique intensities and 289 parameters converged at ω*R*(*F*^2^) = 0.0932 (*R*(*F*) = 0.0415) for 4347 observed reflections with *I* > 2σ(*I*). Crystallographic data for the structure of (+)-(1*R*)-**4b**/(+)-(1*S*)-CSA salt in this paper have been deposited in the Cambridge Crystallographic Data Centre as supplementary publication number CCDC 1580544. Copies of the data can be obtained, free of charge, on application to CCDC, 12 Union Road, Cambridge CB21EZ, UK (fax: +44(0)-1223-336033 or e-mail: deposit@ccdc.cam.ac.uk).

*(−)-(1S) 1-(4-Methoxyphenyl)-2-(1H-pyrazol-1-yl)ethan-1-ol* (**4b**): Yield = 38%. White powder, Mp = 113–114 °C. [α_D_] = −10.0 (*c* 1.0, MeOH) and >99% de (retention time *t_R_* = 52.6 min. using hexane/*i*-PrOH 96:4 *v*/*v* as eluent, flow rate = 0.6 mL/min.). ^1^H NMR (DMSO-*d*_6_) δ = 3.73 (s, 3H, OCH_3_), 4.06–4.30 (m, 2H, H-2, CH_2_, H-2), 4.87 (q, 1H, *J* = 5.6 Hz, CH, H-1), 5.50 (br d, 1H, *J* = 4.5 Hz, OH), 6.16 (t, 1H, *J* = 2.0 Hz, H-4′, Ar), 6.87 (d, 2H, *J* = 8.6 Hz, H-3a, H-5a, Ar), 7.21 (d, 2H, *J* = 8.6 Hz, H-2a, H-6a, Ar), 7.41 (d, 1H, *J* = 1.8 Hz, H-3′, Ar), 7.56 (d, 1H, *J* = 2.2 Hz, H-5′, Ar). ^13^C NMR (DMSO-*d*_6_) δ = 55.0 (OCH_3_), 58.7 (C-2), 71.4 (C-1), 104.6 (C-4′), 113.5 (C-3a, C-5a), 127.2 (C-2a, C-6a), 130.5 C-5′), 134.8 (C-1a), 138.4 (C-3′), 158.5 (C-4a). ES^+^ HRMS, *m*/*z* = 241.0950 found (calculated for C_12_H_14_N_2_O_2_Na [M – H + Na]^+^ requires 241.0953).

*(+)-(1R) 1-(4-Methoxyphenyl)-2-(1H-pyrazol-1-yl)ethan-1-ol* (**4b**): Yield = 41%. White powder, Mp = 110–111 °C. [α_D_] = +10.0 (*c* 1.0, MeOH) and >99% de (retention time *t_R_* = 63.5 min. using hexane/*i*-PrOH 96:4 *v*/*v* as eluent, flow rate = 0.6 mL/min.). ^1^H NMR (DMSO-*d*_6_) δ = 3.73 (s, 3H, OCH_3_), 4.09–4.29 (m, 2H, H-2, CH_2_, H-2), 4.87 (q, 1H, *J* = 5.6 Hz, CH, H-1), 5.50 (br d, 1H, *J* = 4.5 Hz, OH), 6.16 (t, 1H, *J* = 2.0 Hz, H-4′, Ar), 6.87 (d, 2H, *J* = 8.6 Hz, H-3a, H-5a, Ar), 7.22 (d, 2H, *J* = 8.6 Hz, H-2a, H-6a, Ar), 7.41 (d, 1H, *J* = 1.8 Hz, H-3′, Ar), 7.56 (d, 1H, *J* = 2.2 Hz, H-5′, Ar). ^13^C NMR (DMSO-*d*_6_) δ = 55.0 (OCH_3_), 58.7 (C-2), 71.4 (C-1), 104.6 (C-4′), 113.5 (C-3a, C-5a), 127.2 (C-2a, C-6a), 130.5 C-5′), 134.8 (C-1a), 138.4 (C-3′), 158.5 (C-4a). ES^+^ HRMS, *m*/*z* = 241.0950 found (calculated for C_12_H_14_N_2_O_2_Na [M − H + Na]^+^ requires 241.0953).

### 3.2. Biology Section

#### 3.2.1. Reagents

Dimethyl Sulfoxyde (DMSO-Ref D2438) and Thapsigargin (Tg-Ref: T9033) was purchased from Sigma-Aldrich. Corning^®^ Cell-Tak™ was purchased from Beckton Dickinson (Le Pont de Claix, France) (Ref: 354241). Fura-2 QBT™ Calcium Kit was purchased from Molecular Devices (Berkshire, UK) (Ref: R8198).

#### 3.2.2. Cell Culture

The B-cell leukemia cell line PLP [32], which possesses the phenotypic characteristics of B-CLL cells [33], were maintained in RPMI-1640 containing 10% fetal calf serum and antibiotics (1% of penicillin/streptomycin).

#### 3.2.3. Cytosolic Ca^2+^ Protocol

For Ca^2+^ experiments, cells were seeded overnight into four wells plate (Nunc^TM^ multidisches 4 wells flat-bottom-Ref: 176740) at 7 × 10^5^ cells/mL in 1 mL culture medium. Cell suspension was then transfered into 1.5 mL Microtube, pelleted, and loaded with Fura-2 acetoxymethyl ester (Fura-2 QBT™) fluorochrome according to the manufacturer’s protocol. Cell suspension was dispensed onto Corning^®^ Cell-Tak™ pre-coated 96 wells black clear bottom plate (80 µL per wells, density approximatively 5 × 10^4^ cells per well) and incubated 1 h at 37 °C, 5% CO_2_. The Fura-2 QBT™ was aspirated and replaced by an equal volume of free Ca^2+^ Hepes-buffered solution containing (in mM): 135 NaCl, 5 KCl, 1 MgCl_2_, 1 EGTA, 10 Hepes, 10 glucose, pH adjusted at 7.45 with NaOH. The tested compounds or DMSO control solvent solutions were added in wells (4 µL) and incubated for 5 min at 37 °C until evaluating calcium level on FlexStation 3™ Instrument.

#### 3.2.4. Measurement of Intracellular Calcium Levels

Intracellular calcium levels were monitored by using the FlexStation 3™ (Molecular Devices, Berkshire, UK), a fluorescence plate reader measuring time–resolved intracellular Ca^2+^ concentration in a 96-well format with the dual-wavelength fluorescent calcium-sensitive dye Fura-2AM. Dual excitation wavelength capability permits ratiometric measurements of Fura-2AM peak emissions (510 nm) after excitations at 340 (bound to calcium) and 380 nm (unbound to Ca^2+^). Modifications in the 340/380 ratio reflect changes in intracellular-free Ca^2+^ concentrations. The FlexStation 3™ temperature was setting at 37 °C during data acquisition. Thapsigargin (2 µM) solution and Hepes-buffered solution with Ca^2+^ containing (in mM): 135 NaCl, 5 KCl, 1 MgCl_2_, 2 CaCl_2_, 10 Hepes, 10 glucose, pH adjusted at 7.45 with NaOH were added from a 96-well reservoir plate during Calcium Mobilization Assay running at 100 and 750 s, respectively. Experimental setup parameters were optimized (pipette heights, volumes and rate of additions) to minimize disturbance of the cells while ensuring rapid mixing. The data were stored for later analysis by using SoftmaxPro (Molecular Devices), Excel and Graph Pad Prism software (Graph Pad Software, La Jolla, CA, USA).

#### 3.2.5. Measurement of Store-Operated Calcium (SOC) Entry

Intracellular Ca^2+^ stores were depleted with 2 μM Thapsigargin an inhibitor of ER SERCA pumps under Ca^2+^-free conditions to determine the magnitude of intracellular Ca^2+^ release. Next, cells were returned to Ca^2+^-containing Hepes-buffered solution to measure SOCE. The magnitude of SOCE was estimated as the maximal values of normalized F340/F340 ratio following Ca^2+^ re-addition.

#### 3.2.6. Calculation and Data Analysis

Results are expressed as mean ± SEM for each group. The number of experiments for each group is represented as n. Data analysis for functional assays was performed using Softmax Pro and Graph Pad Prism. (Graph Pad): Ca^2+^ concentration variations are estimated using the ratio of peak RFU at 340 and 380 nm (F340/F380) and for each measurement F340/F380 ratio values are normalized to the initial basal ratio before TG addition. Results are expressed in terms of percentage of response or percentage of inhibition compared to the maximum response on control condition. Inhibition is estimated as follow: % Inhibition = (100 − % response). The EC_50_ values were estimated from the dose/response curves (log(inhibitor) vs. amplitude of the Ca^2+^ response) using a regression model (Y = Min + (Max − Min)/(1 + 10^(LogIC50−X)×HillSlope^) with variable slope (Prism5 GraphPad software). Min and Max are the minimal and maximal inhibition obtained. EC_50_ is defined as the concentration of drug required to obtain 50% of inhibitory effect.

## Figures and Tables

**Table 1:** Results for the preparation of 1-phenyl-2-(1*H*-pyrazol-1-yl)ethan-1-one **3a**–**d**, 1-phenyl-2-(1*H*-pyrazol-1-yl)ethan-1-ol **4a**–**d** and (±)-(1*R*, 1*S*) 1-[β-(phenylalkoxy)-phenethyl]-1*H*-pyrazolium hydrochloride **7a**–**e** from phenylalkyl halides **5a**–**d**.

Compound	R ^1^	R ^2^	R ^3^	*n*	R ^4^	Yield (%) ^a^	Overall Yield (%) ^b^
**3a**	H	H	H	-	-	53	-
**3b**	MeO	H	H	-	-	66	-
**3c**	MeO	CF_3_	H	-	-	68	-
**3d**	MeO	CF_3_	CF_3_	-	-	69	-
**4a**	H	H	H	-	-	82	43
**4b**	MeO	H	H	-	-	86	57
**4c**	MeO	CF_3_	H	-	-	94	64
**4d**	MeO	CF_3_	CF_3_	-	-	98	68
**7a**	H	H	H	0	MeO	10	4
**7b**	H	H	H	2	MeO	21	9
**7c**	MeO	H	H	0	MeO	33	19
**7d**	MeO	H	H	2	MeO	18	10
**7e**	MeO	H	H	2	H	33	17
**7f** ^c^	H	H	H	2	H	0	-
**7g** ^d^	MeO	CF_3_	H	0	MeO	0	-
**7h** ^d^	MeO	CF_3_	H	2	MeO	0	-
**7i** ^d^	MeO	CF_3_	H	2	H	0	-
**7j** ^d^	MeO	CF_3_	CF_3_	0	MeO	0	-
**7k** ^d^	MeO	CF_3_	CF_3_	2	MeO	0	-
**7l** ^d^	MeO	CF_3_	CF_3_	2	H	0	-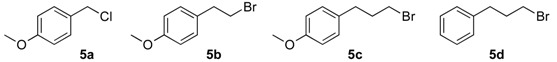							

^a^ Isolated yield after purification; ^b^ Overall yield calculated from compound **3**; ^c^ No crystallization observed after 168 h; ^d^ No crystallization after 336 h.

**Table 2:** Optimization of the reaction conditions for the preparation of the intermediates **6a**–**n**.

Entry	Starting Compound 4	Reagent 5 for *O*-alkylation	KOH (Number of Equiv.)	Reaction Time (h)	Conversion for 6 (%) ^a^	
1	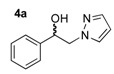	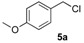	2	48	**6a**	6′
2		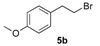	2	48	**6m**	-
3		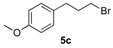	3	48	**6b**	76
4		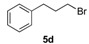	2	48	**6f**	50
5	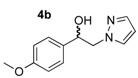	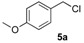	5	72	**6c**	68
6		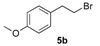	5	72	**6n**	-
7		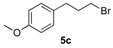	5	72	**6d**	43
8		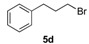	5	72	**6e**	42
9	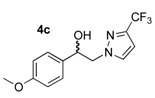	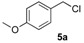	5	72	**6g**	86
10		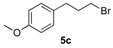	5	72	**6h**	80
11		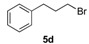	5	72	**6i**	74
12	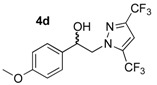	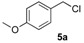	5	72	**6j**	78
13		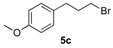	5	72	**6k**	70
14		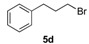	5	72	**6l**	67

^a^ Conversion determined by ^1^H NMR in DMSO-*d*_6_ solution after monitoring of the reaction mixture by TLC on 0.2 mm precoated plates of silica gel 60 F-254 (Merck KGaA, Darmstadt, Germany).

**Table 3:** Products issued from resolution of (±)-(1*R*, 1*S*) 1-(4-methoxyphenyl)-2-(1*H*-pyrazol-1-yl)ethan-1-ol **4b** with (−)-(1*R*)-CSA and (+)-(1*S*)-CSA using the method of “half-quantities”.

Product		[α_D_] ^a^	Retention Time *t_R_* (min.) ^b^	Yield (%) ^e^
(−)-(1*S*)-**4b**/(−)-(1*R*)-CSA	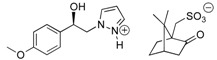	−6	34.0 ^c^	48
(+)-(1*R*)-**4b**/(+)-(1*S*)-CSA	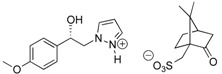	+6	31.5 ^c^	51
(−)-(1*S*)-**4b**	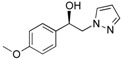	−10	52.6 ^d^	38
(+)-(1*R*)-**4b**	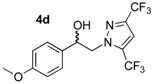	+10	63.5 ^d^	41

^a^ From: *c* 1.0, MeOH; ^b^ Retention time obtained by chiral HPLC analysis using Chiracel OJ-H column (250 × 4.60 mm) and UV detector at 220 nm; ^c^ hexane/*i*-PrOH 94:6 *v*/*v* as eluent with flow rate = 0.8 mL/min; ^d^ hexane/*i*-PrOH 96:4 *v*/*v* as eluent with flow rate = 0.6 mL/min; ^e^ Isolated yield after purification.

**Table 4:** Results for effects of (±)-(1*R*, 1*S*) 1-[β-(phenylalkoxy)-phenethyl]-1*H*-pyrazolium hydrochloride **7a**–**e** on store-operated Ca^2+^ entry with PLP-B lymphocyte. Store-operated Ca^2+^ entry was induced by depletion of Endoplasmic Reticulum Ca^2+^ stores (ER Ca^2+^ stores) with thapsigargin (Tg) in a Ca^2+^-free medium and measured following the addition of 1.8 mM CaCl_2_ in the extracellular medium.

Compound 7	Concentration of 7 (μM)	Effect of 7 on Amplitude of SOCE Peak ^a^	IC_50_ (μM) ^b^
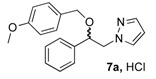	100	−44.4	102
	50	−29.0	
	10	−14.5	
	1	−12.0	
	0.1	−24.5	
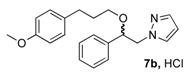	100	−83.0	34
	50	−65.0	
	10	−14.8	
	1	−9.3	
	0.1	−5.6	
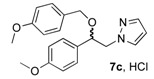	100	−53.1	60
	50	−46.0	
	10	−2.2	
	1	−7.1	
	0.1	7.6	
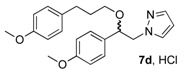	100	−87.5	25
	50	−81.8	
	10	−0.3	
	1	−10.7	
	0.1	−0.3	
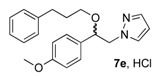	100	−81.7	48
	50	−51.1	
	10	−5.4	
	1	−3.6	
	0.1	−11.3	
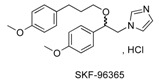	100	−58.7	60
	50	−44.3	
	10	−15.4	
	1	−26.4	
	0.1	−3.4	

^a^ % of inhibition (−) or activation. Controlled with DMSO 100%; ^b^ IC_50_ expressed in μM are the average of three assays, ±0.5 μM.

## Supplementary Materials

Supplementary materials can be found at http://www.mdpi.com/1422-0067/19/3/856/s1.
