# Supplementary material for: Preliminary Structure-Activity Relationship (SAR) of a Novel Series of Pyrazole SKF-96365 Analogues as Potential Store-Operated Calcium Entry (SOCE) Inhibitors
Source: Int J Mol Sci. 2018 Mar 14;19(3):856. doi: 10.3390/ijms19030856 (PMC5877717; doi:10.3390/ijms19030856)

SUPPLEMENTARY MATERIALS

Preliminary SAR of a novel series of pyrazole SKF-96365 analogues as potential Store-Operated Calcium Entry (SOCE) inhibitors

Camille D. Dago[1,2], Paul Le Maux[1], Thierry Roisnel[3], Christophe Brigaudeau[4,5], Yves-Alain Bekro[2], Olivier Mignen[4,5,*] and Jean-Pierre Bazureau[1,6,*]

[1] Institut des Sciences Chimiques de Rennes (ISCR), UMR CNRS 6226, groupe CORINT, Université de Rennes 1 (UR1), Campus de Beaulieu, Bât. 10A, 263 Avenue du Général Leclerc, CS 74205, 35042 Rennes Cedex, France

[2] Laboratoire de Chimie Bio Organique et de Substances Naturelles (LCBOSN), Université Nangui Abrogoua (UNA), BP 802, Abidjan, Côte d'Ivoire

[3] Institut des Sciences Chimiques de Rennes (ISCR), UMR CNRS 6226, Centre de Diffractométrie X (cdifx), Université de Rennes 1 (UR1), Campus de Beaulieu, Bât. 10B, 263 Avenue du Général Leclerc, CS 74205, 35042 Rennes Cedex, France

[4] Laboratoire Canalopathies & Signalisation Calcique, Inserm U1277, Université de Bretagne Occidentale (UBO), 22 Avenue Camille Desmoulins, 29200 Brest Cedex, France

[5] CalciScreen platform, Université de Bretagne Occidentale (UBO), 22 Avenue Camille Desmoulins, 29200 Brest Cedex, France

[6] S2Wave platform SFS ScanMAT, Université de Rennes 1 (UR1), Campus de Beaulieu, Bât. 10A, 263 Avenue du Général Leclerc, CS 74205, 35042 Rennes Cedex, France

Fax: +33 (0)223 236 374. Phone: +33(0)2 223 236 603.
E-mail: jean-pierre.bazureau@univ-rennes1.fr

Part 1:

- ^{1}H NMR in DMSO-d_6 solution of isolated (*1R, 1S*) 1-[β-(phenylalkoxy)-phenethyl]-1*H*-pyrazolium hydrochloride (**7a-e**)

- ^{13}C NMR in DMSO-d_6 solution of isolated (*1R, 1S*) 1-[β-(phenylalkoxy)-phenethyl]-1*H*-pyrazolium hydrochloride (**7a-e**)

- ^{1}H NMR in DMSO-d_6 solution of (*1R, 1S*) 1-[β-((4-methoxybenzyl)oxy)-phenethyl]-1*H*-pyrazolium hydrochloride (**7a**)

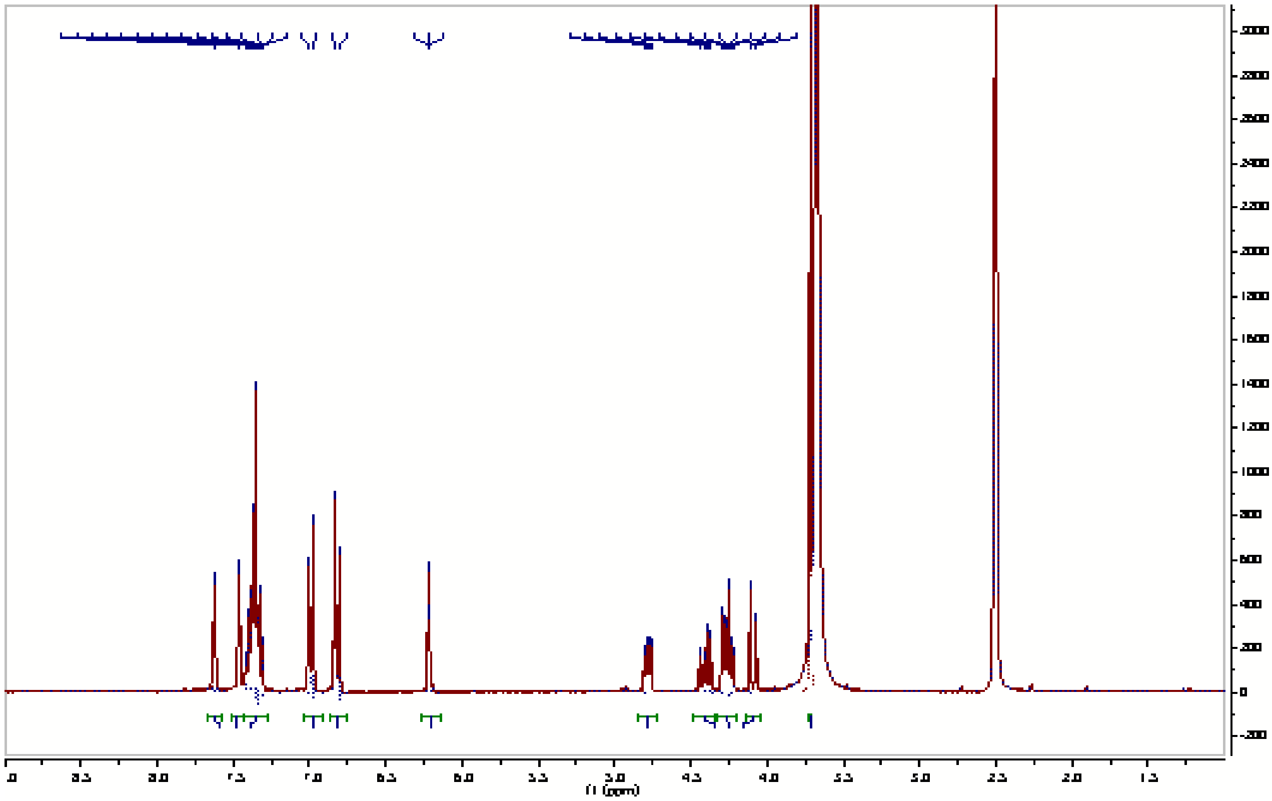

- ^{13}C NMR in DMSO-d_6 solution of (*1R, 1S*) 1-[β-((4-methoxybenzyl)oxy)-phenethyl]-1*H*-pyrazolium hydrochloride (**7a**)

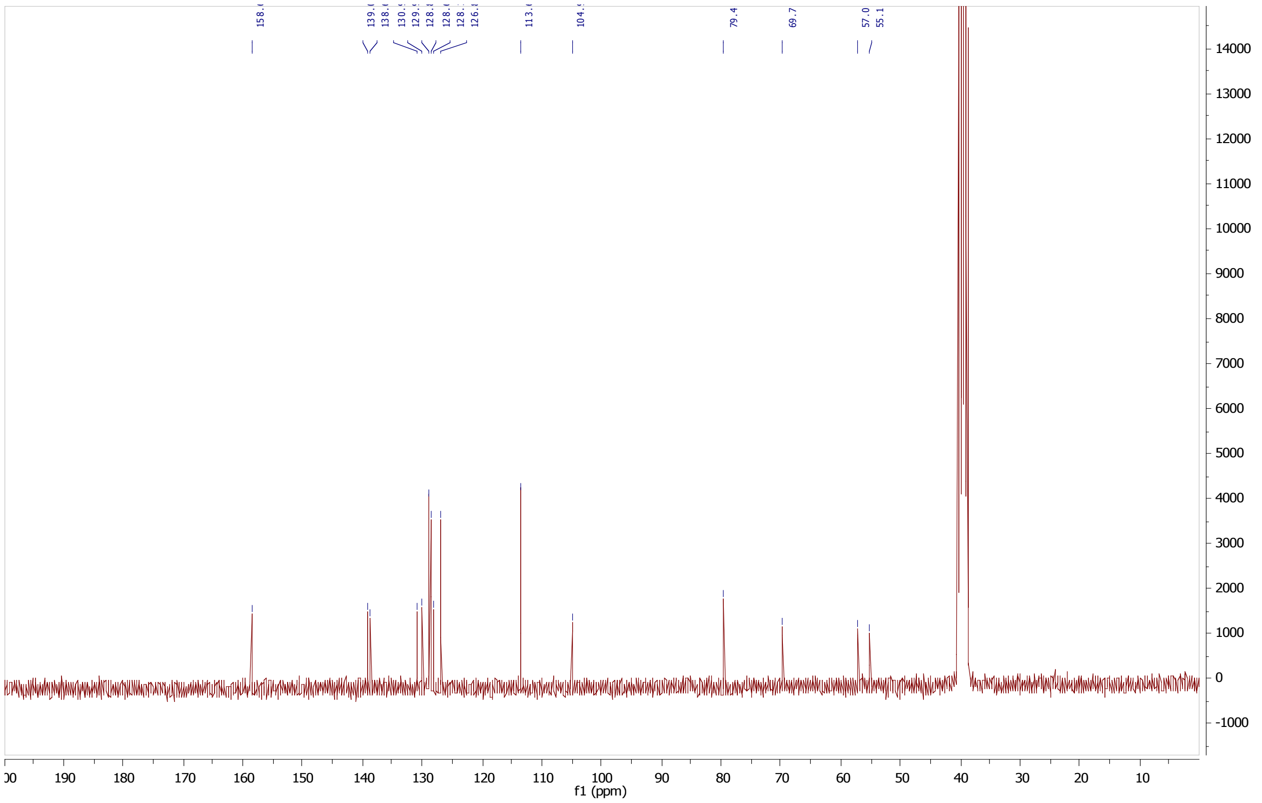

- ^{1}H NMR in DMSO-d_6 solution of (*1R, 1S*) 1-[β-((3-(4-methoxyphenyl)propyl)oxy)-phenethyl]-1*H*-pyrazolium hydrochloride (**7b**)

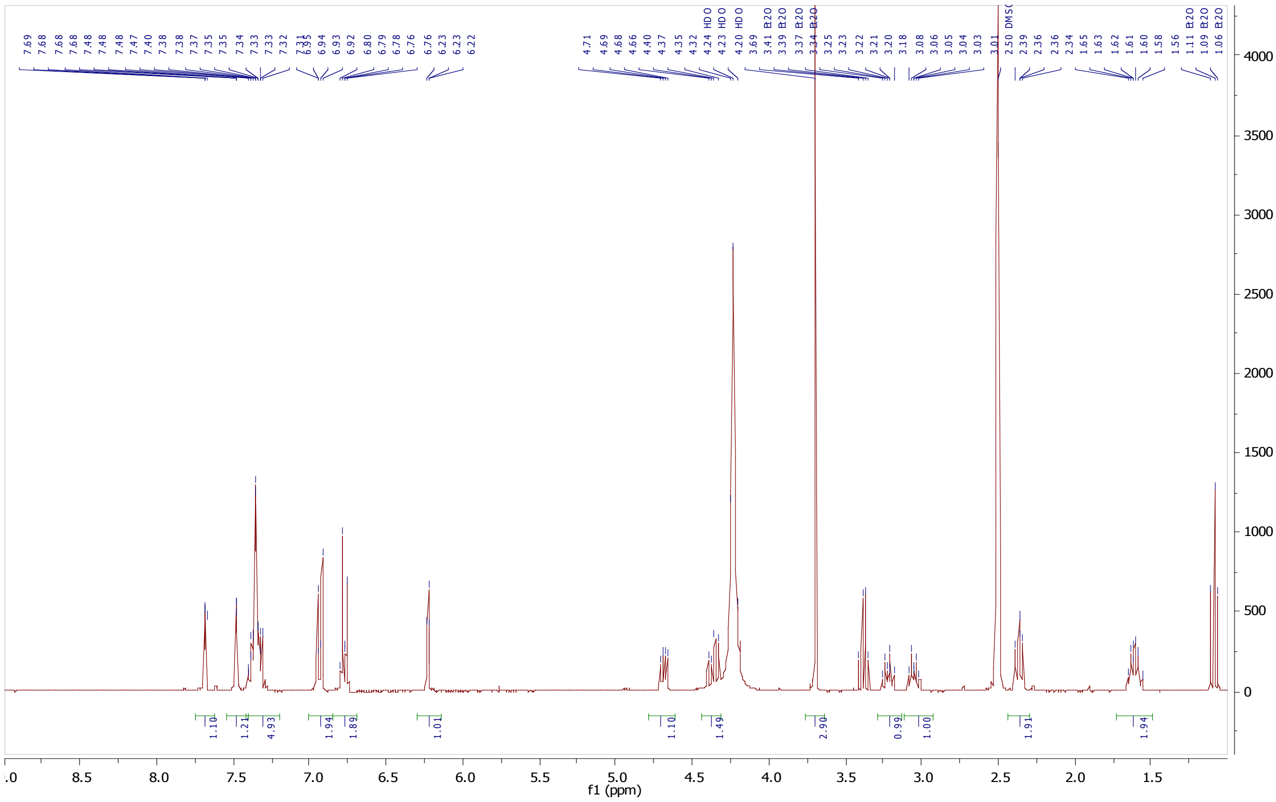

- ^{13}C NMR in DMSO-d_6 solution of (*1R, 1S*) 1-[β-((3-(4-methoxyphenyl)propyl)oxy)-phenethyl]-1*H*-pyrazolium hydrochloride (**7b**)

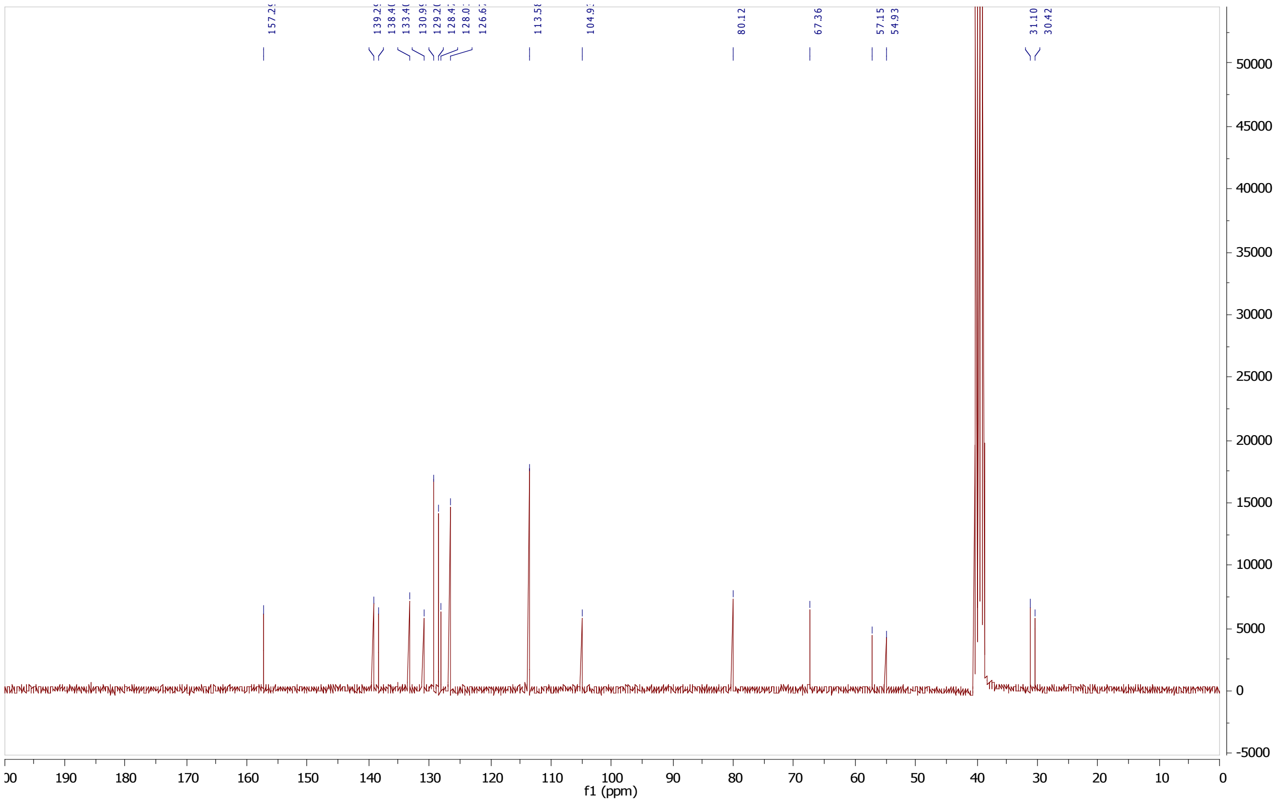

- ^{1}H NMR in DMSO-d_6 solution of (*1R, 1S*) 1-[β-((4-methoxybenzyl)oxy)-(4-methoxyphenethyl]-1*H*-pyrazolium hydrochloride (**7c**)

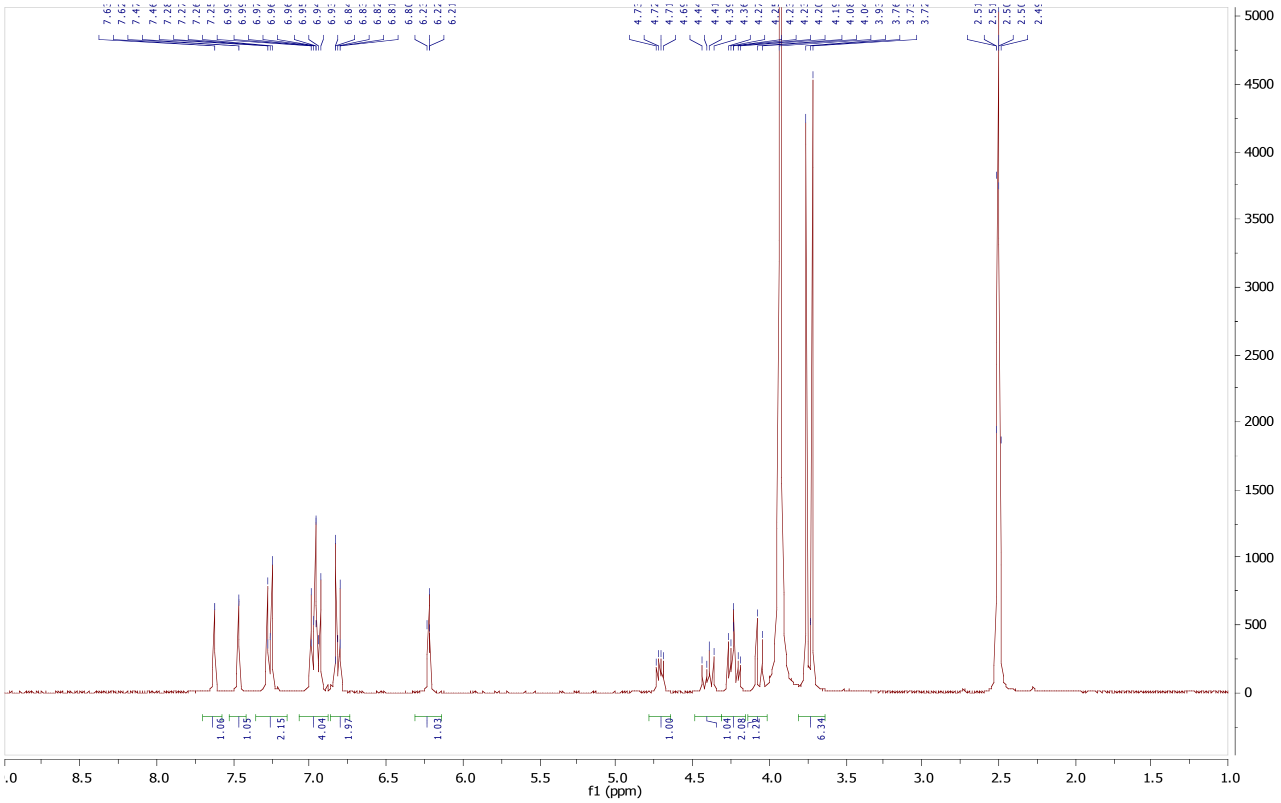

- ^{13}C NMR in DMSO-d_6 solution of (*1R, 1S*) 1-[β-((4-methoxybenzyl)oxy)-(4-methoxyphenethyl]-1*H*-pyrazolium hydrochloride (**7c**)

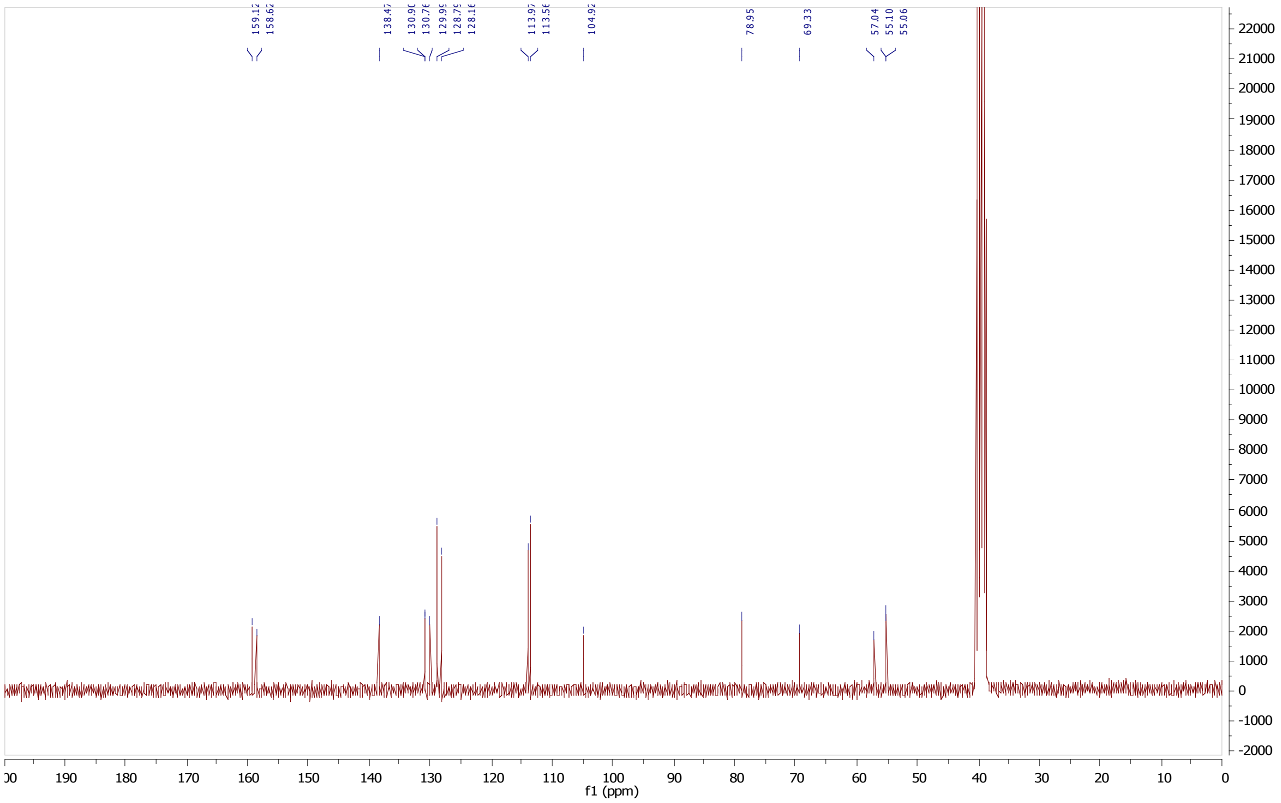

- ^{1}H NMR in DMSO-d_6 solution of (*1R, 1S*) 1-[β-((4-methoxyphenyl)-(3-(4-methoxyphenyl)propoxyethyl)]-1*H*-pyrazolium hydrochloride (**7d**)

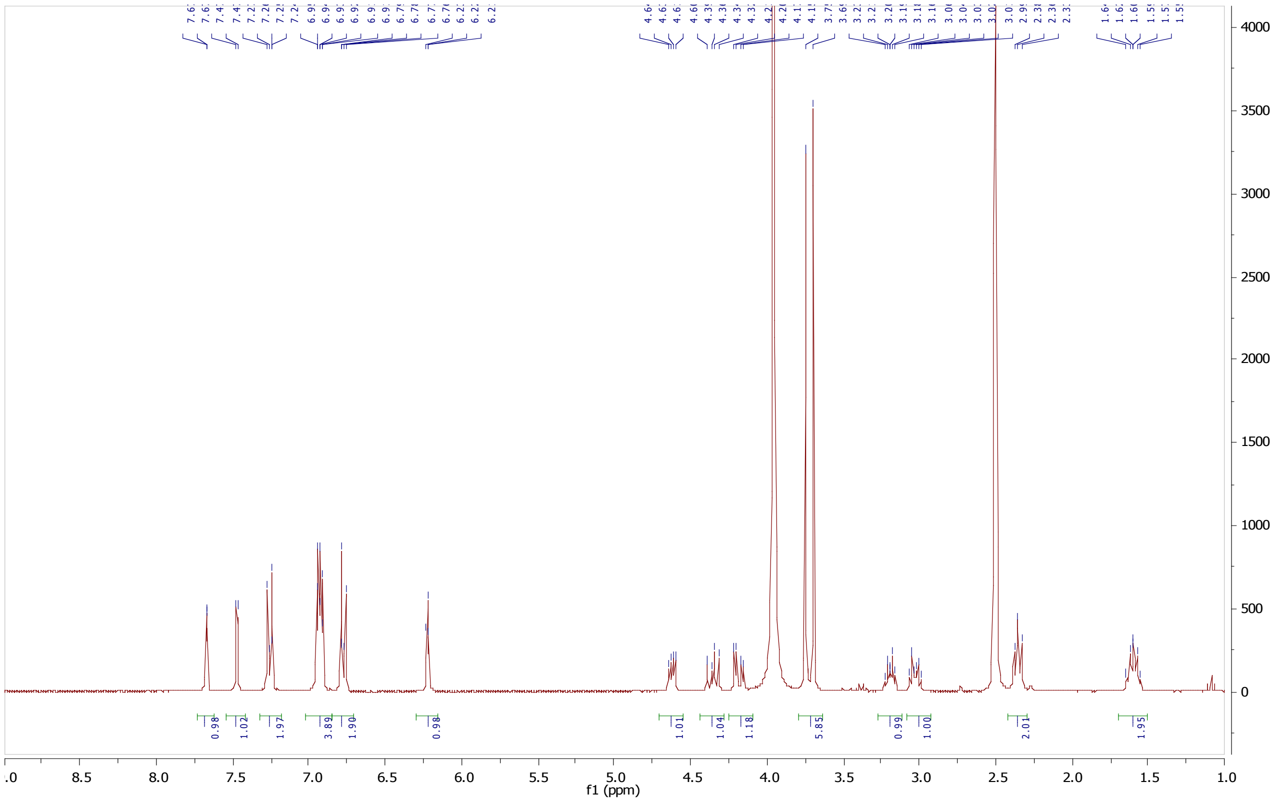

- ^{13}C NMR in DMSO-d_6 solution of (*1R, 1S*) 1-[β-((4-methoxyphenyl)-(3-(4-methoxyphenyl)propoxyethyl)]-1*H*-pyrazolium hydrochloride (**7d**)

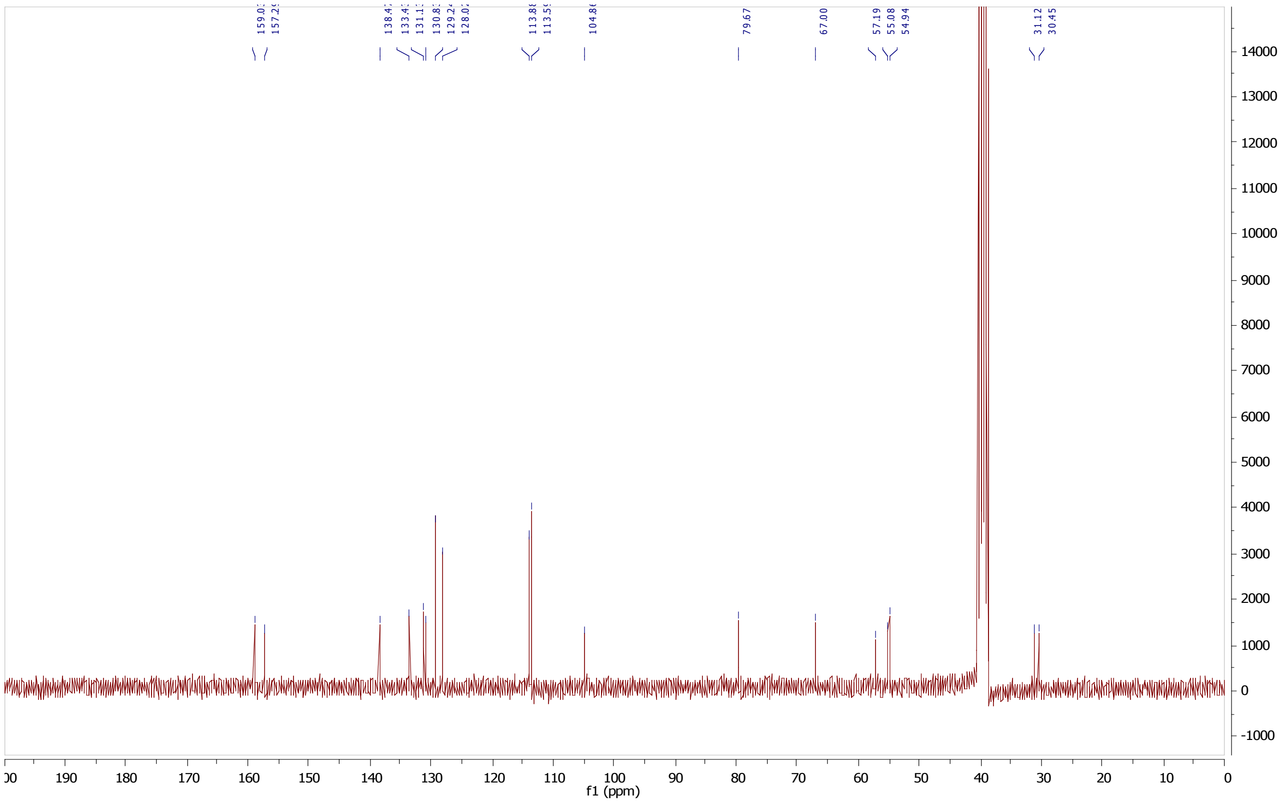

- ^{1}H NMR in DMSO-d_6 solution of (*1R, 1S*) 1-[β-((4-methoxyphenyl)-(3-phenylpropoxyethyl)]-1*H*-pyrazolium hydrochloride (**7e**)

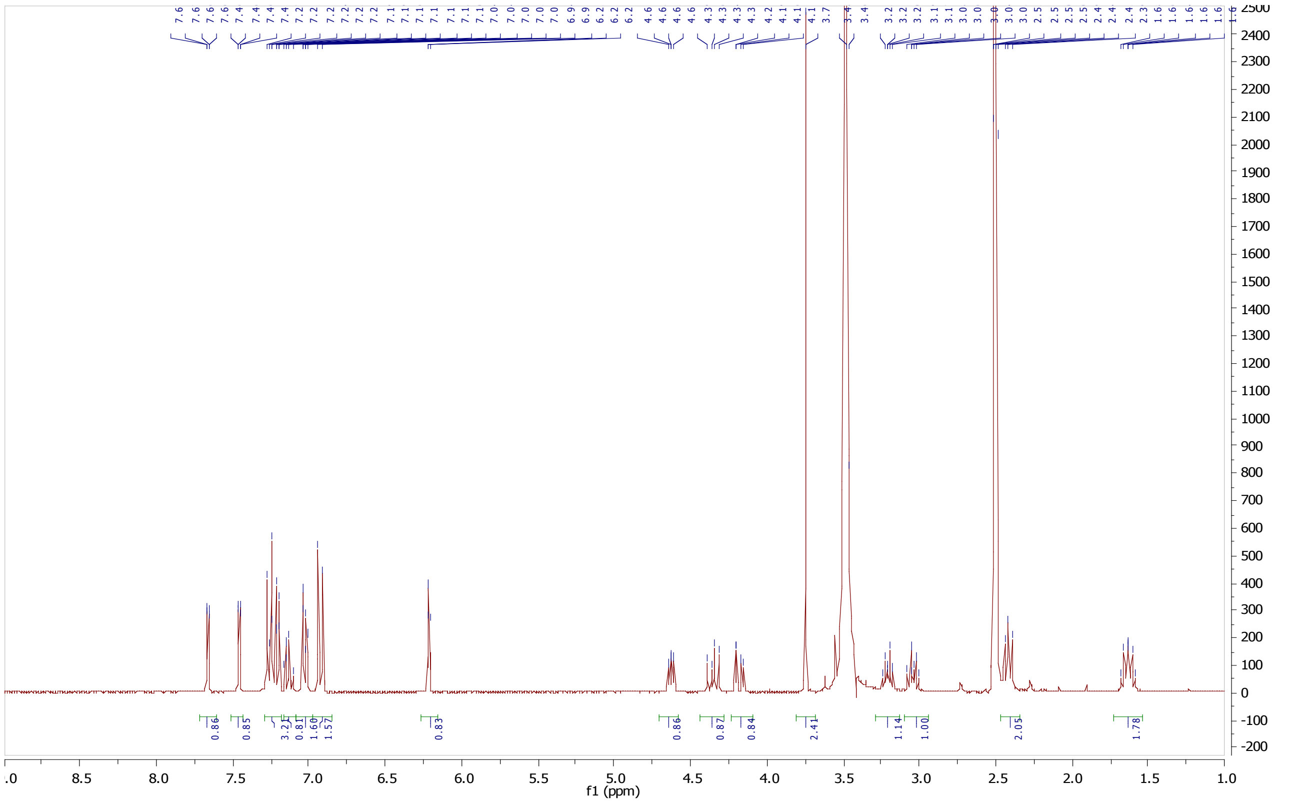

- ^{13}C NMR in DMSO-d_6 solution of (*1R, 1S*) 1-[β-((4-methoxyphenyl)-(3-phenylpropoxyethyl)]-1*H*-pyrazolium hydrochloride (**7e**)

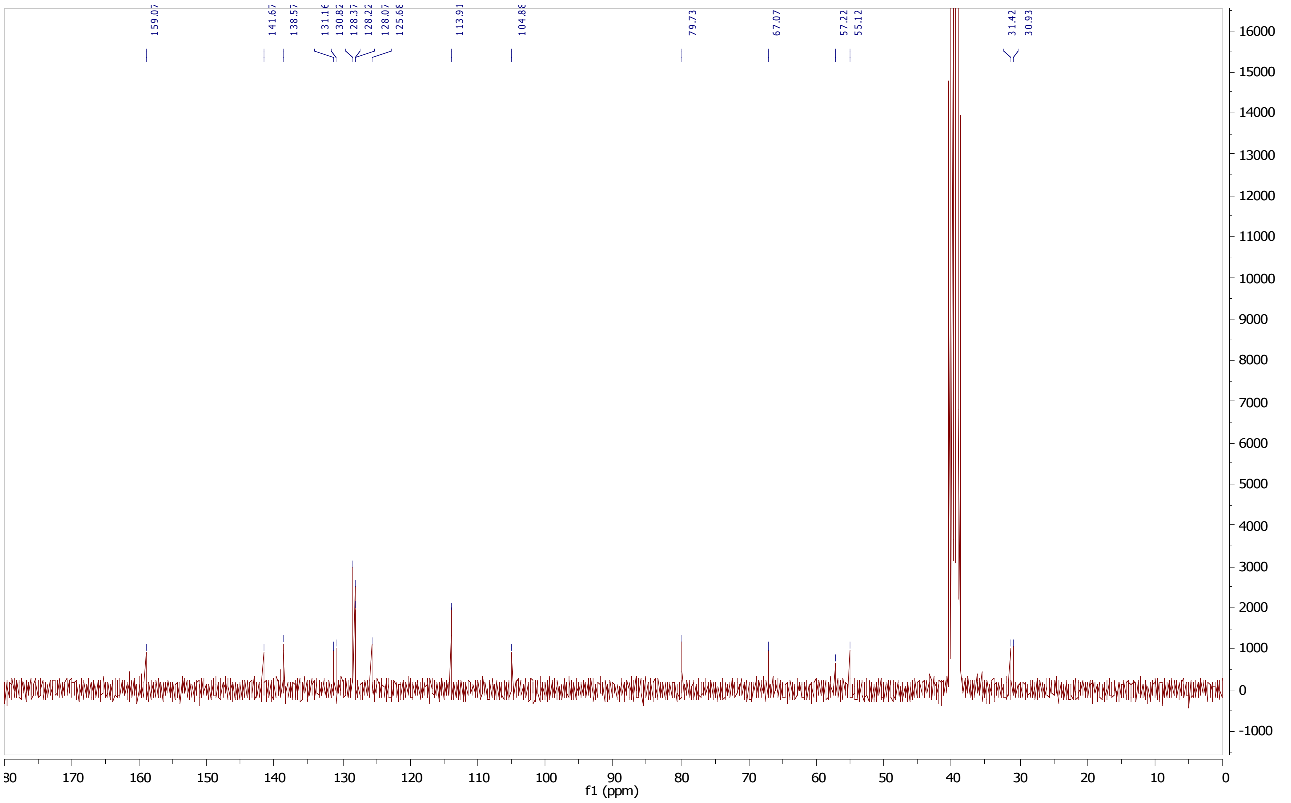

SUPPLEMENTARY MATERIALS

Preliminary SAR of a novel series of pyrazole SKF-96365 analogues as potential Store-Operated Calcium Entry (SOCE) inhibitors

Camille D. Dago[1,2], Paul Le Maux[1], Thierry Roisnel[3], Christophe Brigaudeau[4,5], Yves-Alain Bekro[2], Olivier Mignen[4,5,*] and Jean-Pierre Bazureau[1,6,*]

[1] Institut des Sciences Chimiques de Rennes (ISCR), UMR CNRS 6226, groupe CORINT, Université de Rennes 1 (UR1), Campus de Beaulieu, Bât. 10A, 263 Avenue du Général Leclerc, CS 74205, 35042 Rennes Cedex, France

[2] Laboratoire de Chimie Bio Organique et de Substances Naturelles (LCBOSN), Université Nangui Abrogoua (UNA), BP 802, Abidjan, Côte d'Ivoire

[3] Institut des Sciences Chimiques de Rennes (ISCR), UMR CNRS 6226, Centre de Diffractométrie X (cdifx), Université de Rennes 1 (UR1), Campus de Beaulieu, Bât. 10B, 263 Avenue du Général Leclerc, CS 74205, 35042 Rennes Cedex, France

[4] Laboratoire Canalopathies & Signalisation Calcique, Inserm U1277, Université de Bretagne Occidentale (UBO), 22 Avenue Camille Desmoulins, 29200 Brest Cedex, France

[5] CalciScreen platform, Université de Bretagne Occidentale (UBO), 22 Avenue Camille Desmoulins, 29200 Brest Cedex, France

[6] S2Wave platform SFS ScanMAT, Université de Rennes 1 (UR1), Campus de Beaulieu, Bât. 10A, 263 Avenue du Général Leclerc, CS 74205, 35042 Rennes Cedex, France

Fax: +33 (0)223 236 374. Phone: +33(0)2 223 236 603.

E-mail: jean-pierre.bazureau@univ-rennes1.fr

Part 2:

- Chiral HPLC chromatograms obtained for resolution of (±)-(1*R*, 1*S*) 1-(4-methoxyphenyl)-2-(1*H*-pyrazol-1-yl)ethan-1-ol **4b** with (-)-(1*R*)-CSA and (+)-(1*S*)-CSA using the method of "half-quantities"
- Chiral HPLC chromatogram obtained for racemic (*1R, 1S*) 1-[β-((4-methoxyphenyl)-(3-(4-methoxyphenyl)propoxyethyl)]-1*H*-pyrazolium hydrochloride (**7d**):

- Chiral HPLC analysis obtained for diastereoisomer (-)-(1*S*)-**4b**/(-)-(1*R*)-CSA:

Column: Chiracel OJ-H (250 x 4.60 mm)

Detector: UV, 220 nm

Eluent: hexane/*i*-PrOH (94:6 v/v)

Flow rate: 0.8 mL/min.

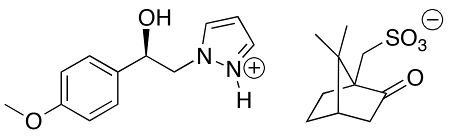

Retention time = 34.0 min.

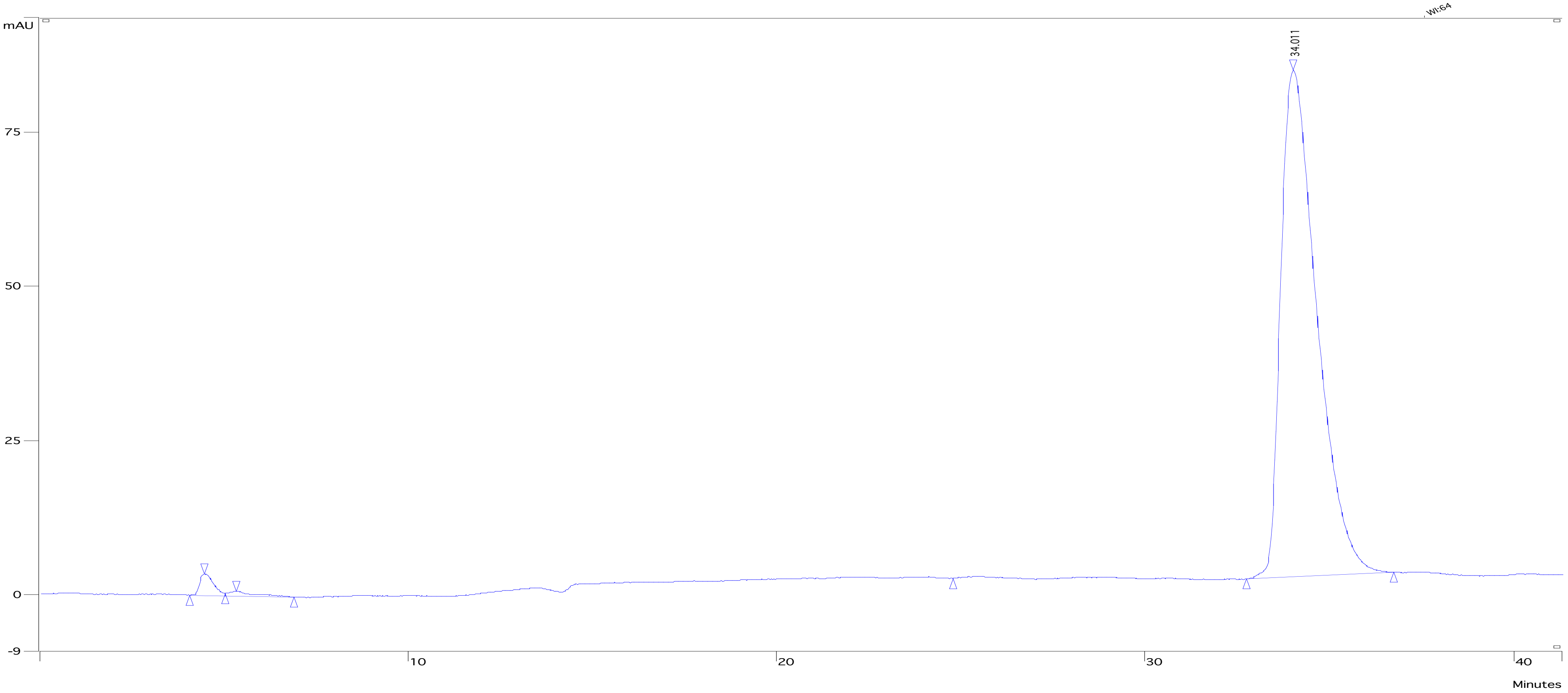

- Chiral HPLC analysis obtained for diastereoisomer (+)-(1*R*)-**4b**/(+)-(1*S*)-CSA:

Column: Chiracel OJ-H (250 x 4.60 mm)

Detector: UV, 220 nm

Eluent: hexane/*i*-PrOH (94:6 v/v)

Flow rate: 0.8 mL/min.

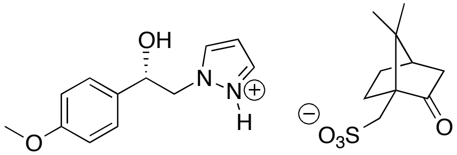

Retention time = 31.5 min.

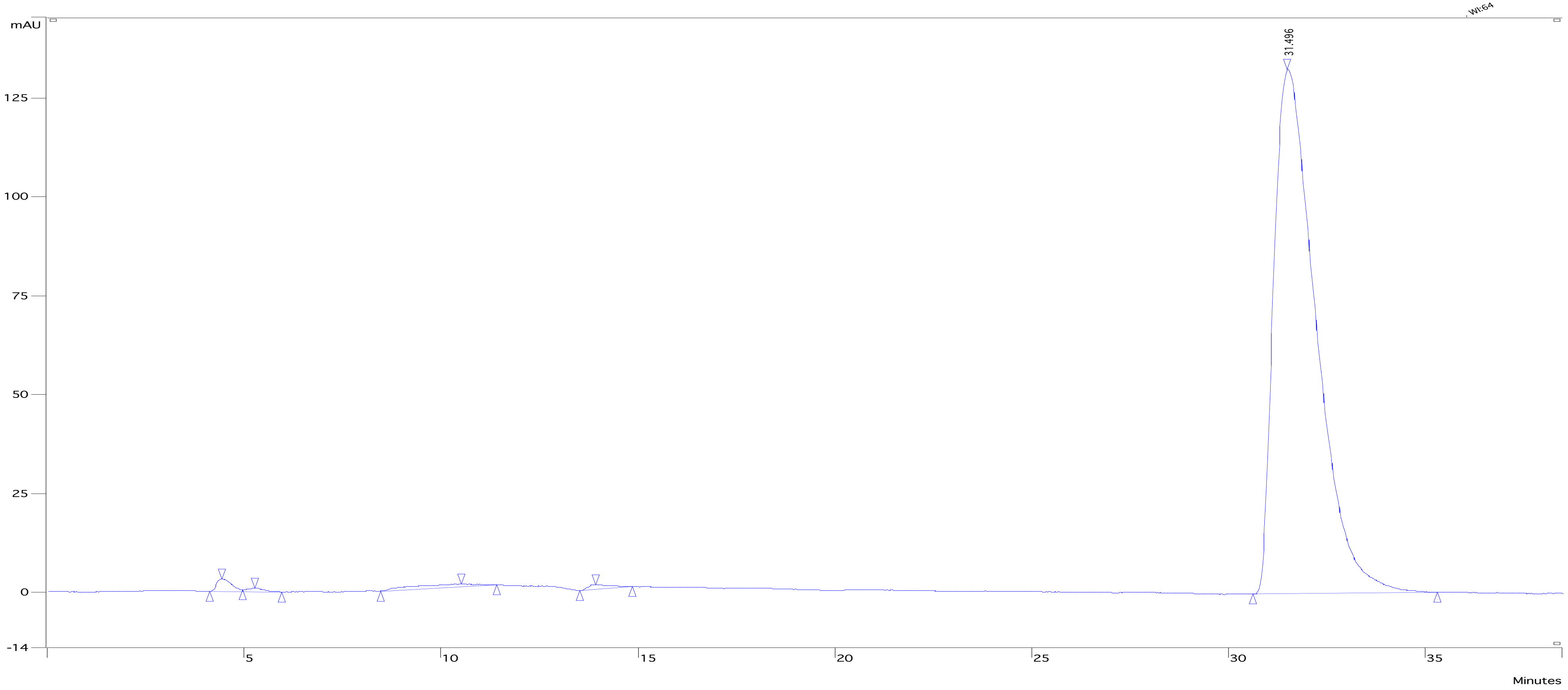

- Chiral HPLC analysis obtained for enantiomer (-)-(1*S*)-**4b**:

Column: Chiracel OJ-H (250 x 4.60 mm)

Detector: UV, 220 nm

Eluent: hexane/*i*-PrOH (94:6 v/v)

Flow rate: 0.6 mL/min.

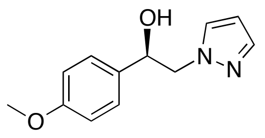

Retention time = 52.6 min.

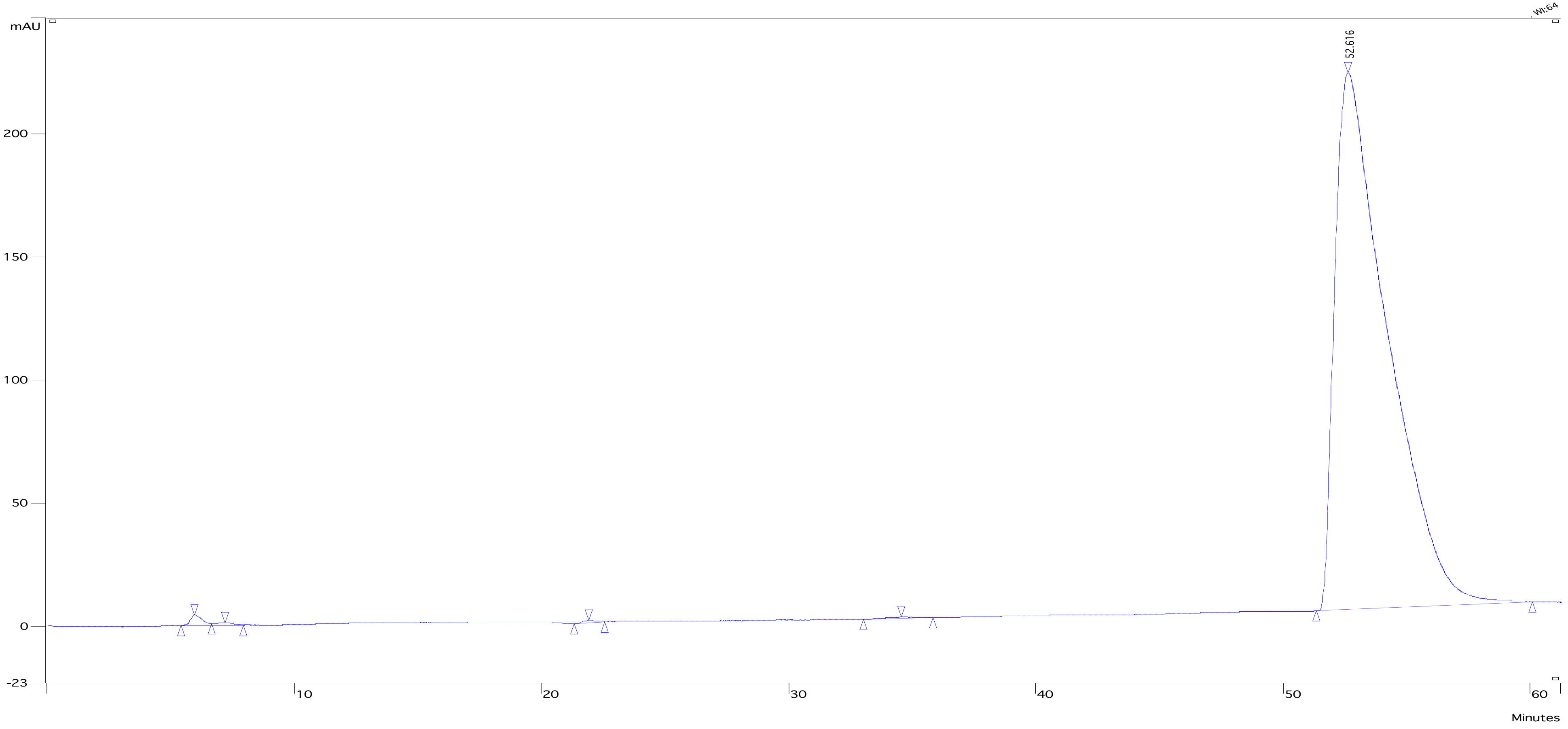

- Chiral HPLC analysis obtained for enantiomer (+)-(1*R*)-**4b**:

Column: Chiracel OJ-H (250 x 4.60 mm)

Detector: UV, 220 nm

Eluent: hexane/*i*-PrOH (94:6 v/v)

Flow rate: 0.6 mL/min.

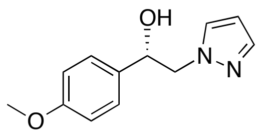

Retention time = 63.5 min.

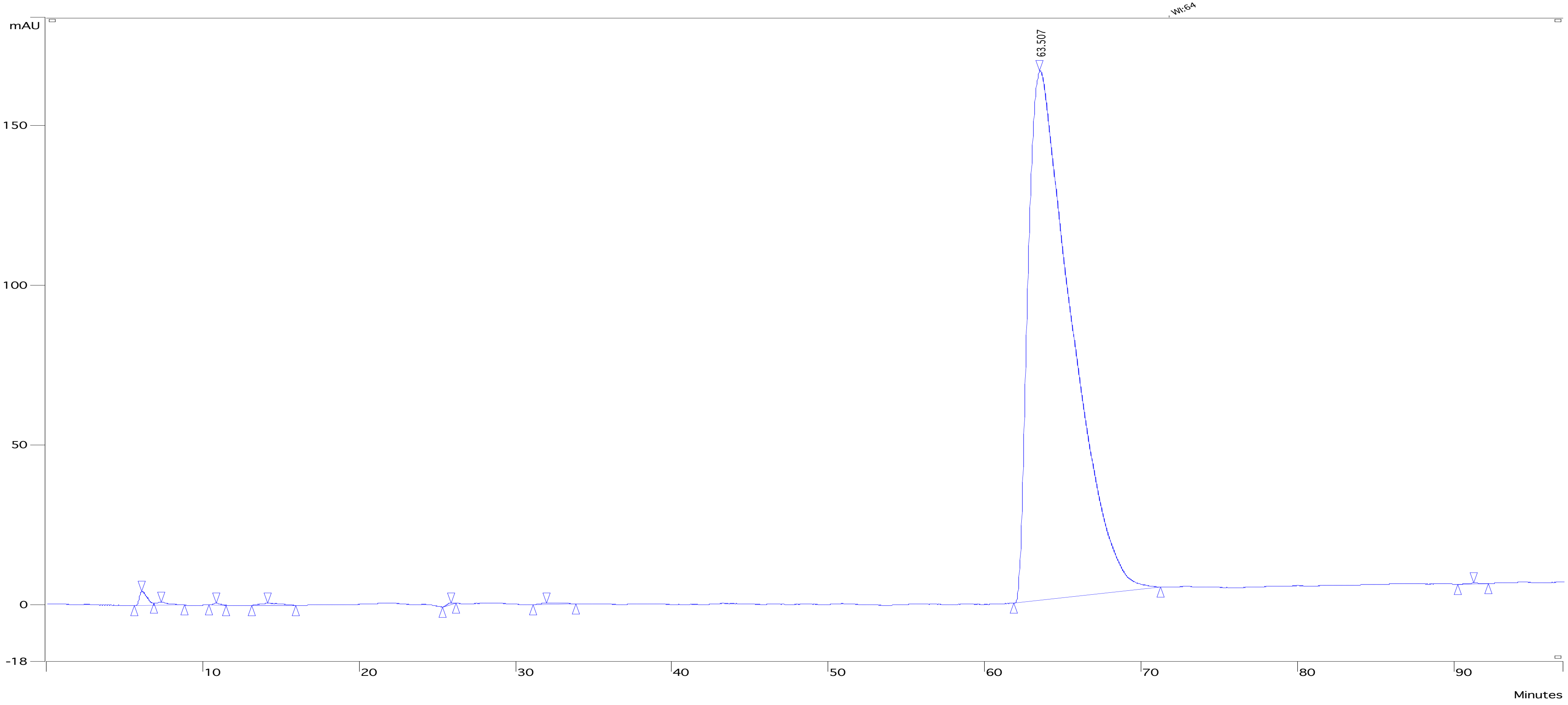

Supplement: Supplementary file 1 [file ijms-19-00856-s001.pdf]
